# A Signaling Factor Linked to Toxoplasma gondii Guanylate Cyclase Complex Controls Invasion and Egress during Acute and Chronic Infection

**DOI:** 10.1128/mbio.01965-22

**Published:** 2022-10-06

**Authors:** Shu Ye, Matteo Lunghi, Dominique Soldati-Favre

**Affiliations:** a Department of Microbiology and Molecular Medicine, Faculty of Medicine, University of Geneva, Geneva, Switzerland; Rutgers—New Jersey Medical School

**Keywords:** *Toxoplasma gondii*, signaling, guanylate cyclase, invasion, egress, bradyzoite, tissue cyst, apicomplexan parasites, chronic infection

## Abstract

Toxoplasma gondii is an intracellular apicomplexan parasite that relies on cyclic GMP (cGMP)-dependent signaling to trigger timely egress from host cells in response to extrinsic and intrinsic signals. A guanylate cyclase (GC) complex, conserved across the Apicomplexa, plays a pivotal role in integrating these signals, such as the key lipid mediator phosphatidic acid and changes in pH and ionic composition. This complex is composed of an atypical GC fused to a flippase-like P4-ATPase domain and assembled with the cell division control protein CDC50.1 and a unique GC organizer (UGO). While the dissemination of the fast-replicating tachyzoites responsible for acute infection is well understood, it is less clear if the cyst-forming bradyzoites can disseminate and contribute to cyst burden. Here, we characterized a novel component of the GC complex recently termed signaling linking factor (SLF). Tachyzoites conditionally depleted in SLF are impaired in microneme exocytosis, conoid extrusion, and motility and hence unable to invade and egress. A stage-specific promoter swap strategy allowed the generation of SLF- and GC-deficient bradyzoites that are viable as tachyzoites but show a reduction in cyst burden during the onset of chronic infection. Upon oral infection, SLF-deficient cysts failed to establish infection in mice, suggesting SLF’s importance for the natural route of T. gondii infection.

## INTRODUCTION

Toxoplasma gondii is an obligate intracellular parasite that belongs to the phylum Apicomplexa and is a ubiquitous opportunistic pathogen infecting humans and animals. It is estimated that around one-third of the global human population is infected by T. gondii (1). Persistent chronic infection caused by encysted bradyzoites is associated with neuropsychiatric and behavioral disorders in the host (2). It is a life-threatening disease in immunosuppressed individuals due to the recrudescence of infection by the bradyzoites’ conversion to the fast-growing tachyzoites (3). Moreover, oral ingestion of tissue cysts is a key source of T. gondii infection. Bradyzoites are therefore of great relevance for pathogenesis and transmission of toxoplasmosis.

During acute infection, the dissemination of parasites in the host is driven by the rapid expansion of the tachyzoite. Their dissemination involves a lytic cycle, which consists of invasion, replication, and egress from infected cells. Parasite invasion and egress are powered by a gliding motility (4). Exocytosis of secretory organelles termed micronemes and an apico-basal flux of filamentous actin (F-actin) generated by actomyosin machinery are two prerequisites for parasite gliding motility (5–7). Recently, the extrusion of the conoid, an apical dynamic macromolecular complex, was shown to guide flux of F-actin along parasite pellicular space (8).

The above complex cascade of events governing parasite egress and invasion is underpinned by a cyclic GMP (cGMP)-protein kinase G (PKG) signaling pathway. Following invasion, tachyzoites reside in a nonfusogenic parasitophorous vacuole (PV), where they replicate safely. Synchronous division and egress are allowed by a cytoplasmic connection, termed the residual body (RB) (9). Tachyzoites naturally egress at around 48 h postinfection in response to accumulation of intravacuolar phosphatidic acid (PA) produced by diacylglycerol kinase 2 (DGK2) (10). Alternatively, parasites can escape from unhealthy host cells at any time of their replication by sensing external stimuli, such as vacuolar acidification (11) and a drop in K^+^ (12). These stimuli are integrated by a guanylate cyclase (GC) complex (10, 13–15). Elevation of GC-produced cGMP activates PKG (16), which presumably promotes the formation of phosphatidylinositol 4,5-bisphosphate [PI_(4,5)_P_2_]. The hydrolysis of PI_(4,5)_P_2_ by PI-phospholipase C (PI-PLC) produces two intracellular mediators: diacylglycerol (DAG) and inositol triphosphate (IP_3_) (17, 18). DAG is converted to PA by diacylglycerol kinase 1 (DGK1) at the parasite periphery, presumably in the inner leaflet of the parasite plasma membrane. The enriched PA is then sensed apically by acylated-pleckstrin-homology protein (APH), which is anchored on the surface of microneme organelles, leading to microneme exocytosis (18, 19). Concurrently, IP_3_ mobilizes internal calcium (Ca^2+^) via a type of signaling not identified to date to activate calcium-dependent protein kinases (CDPKs) involved in the control of microneme secretion, conoid extrusion, and activation of the actomyosin system (5, 20). Treatment with compound 1 (C1), an inhibitor of PKG, or conditional knockdown of CDPK1 blocks microneme exocytosis, conoid extrusion, and the production of an apico-basal flux of F-actin (5, 21). Additionally, CDPK3 was shown to phosphorylate the myosin motor and control microneme secretion in intracellular parasites, regulating calcium-dependent egress (22, 23).

In T. gondii, the GC is fused to a flippase-like P4-ATPase and is found associated with the cell division control 50.1 (CDC50.1) and a unique GC organizer (UGO) (10). This large molecular complex is localized at the apical and basal pole of the parasite plasma membrane. GC catalyzes the synthesis of cGMP, which is degraded to GMP under the control of phosphodiesterases (PDEs) (24–26). Conditional depletion of GC impairs tachyzoite motility, invasion, and egress. Depletion of CDC50.1, which serves as chaperone for the P4-ATPase domain of GC, leads to mislocalization of GC that fails to respond to PA. In addition to its role in GC trafficking, UGO is also essential for GC activity, either by contributing to the folding of GC catalytic domains or by GC activation through the sensing of stimuli (10). Despite the pivotal role of GC, the composition and contribution of the complex in integrating multiple parasite- and host-derived stimuli remain elusive. It is also unknown whether the GC needs additional partners for signal perception and transduction.

In contrast to tachyzoites, our knowledge about bradyzoites’ lytic cycle and dissemination is partial. During acute infection, tachyzoites disseminate throughout the host and traverse the blood-brain barrier (BBB). Three major models have been proposed to explain how T. gondii crosses the BBB and accesses the central nervous system: (i) free parasites use gliding motility to transmigrate through the endothelial adherent junctions, (ii) tachyzoites invade and hijack natural immune cells, which can then migrate across the BBB, and (iii) the brain endothelial cells (ECs) are infected and lysed by parasites, leading to parasite release into the brain parenchyma (27). The adaptive immune response of the host starts with the appearance of anti-*Toxoplasma* IgM antibodies 1 week postinfection and becomes efficient with the presence of IgG after 2 weeks (28). Under immune pressure, chronic infection is initially established by tachyzoite conversion into bradyzoites. Based on the mouse infection model, the cyst burden in brain remains stable between weeks 3 and 8 postinfection, which can be explained by the clearance of tachyzoites and the surveillance on bradyzoites with efficient immune modulation of the host (29). The period of the first 2 weeks following inoculation corresponds to the acute phase of infection. During the early phase of chronic infection (between weeks 2 and 3), an increase in cyst burden is expected. It is unclear whether the newly formed tissue cysts are mainly derived from the tachyzoites’ brain colonization or if bradyzoites released from ruptured cysts are invasive and contribute to cyst dissemination. Also, during natural infection via ingestion of cysts, it is not known if the GC complex is critical in sensing local signals to trigger motility and invasion.

Bradyzoites are surrounded by a heavily glycosylated cyst wall which is considerably remodeled from the tachyzoite’s PV membrane (30, 31). Slow asynchronous bradyzoite replication has been observed (9, 29) leading to tissue cysts containing up to several thousands of bradyzoites, depending on cyst maturity, and consequently to a broad range of cyst diameter (32). These observations could indicate a dynamic nature of bradyzoites. Recent work demonstrated that calcium signaling controls bradyzoite gliding motility (33). Upon oral ingestion, bradyzoites are released from tissue cysts and invade intestinal epithelium cells (34). Overall, these data indicate that bradyzoites might be using the same signaling and motility machinery as tachyzoites.

In the present study, we functionally characterized the *T. gondii* signaling linking factor (TgSLF), an additional essential component of the GC complex that governs tachyzoites’ motility, invasion, and egress. Based on a promoter swap strategy, bradyzoite stage-specific SLF and GC knockdown strains were generated. By comparing the cyst burdens at different time points of mice infected with the promoter swap strains, we could report a modest contribution of SLF and GC to the establishment of chronic infection and no apparent role in maintenance of cyst numbers over the course of infection. Instead, we reveal a crucial role of SLF at the time of natural oral infection with tissue cysts.

## RESULTS

### SLF is an interacting partner of the guanylate cyclase complex.

**FIG 1:**
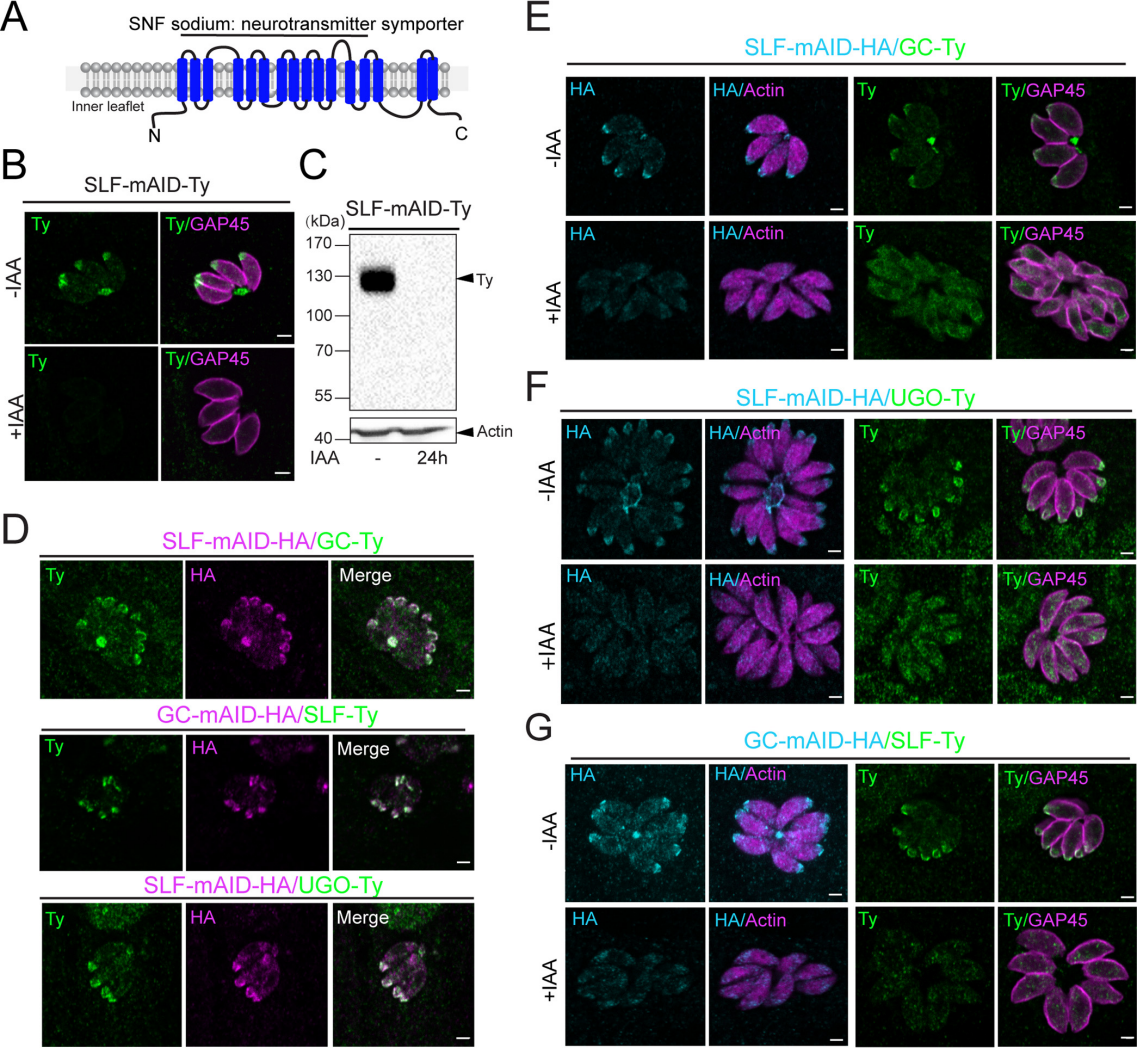
SLF is a partner of the GC complex. (A) Predicted SLF protein structure with 16 TMs (blue cylinders) and cytoplasmic amino termini and carboxyl termini. The region of 1 to 13 TMs is annotated as a sodium neurotransmitter symporter (SNF). (B) Indirect immunofluorescence assay (IFA) showing the apical tip and basal pole localization of endogenously C-terminally mAID-Ty-tagged SLF, which is not detectable in the presence of indole-3-acetic acid (IAA) for 24 h. GAP45, parasite pellicle. (C) Western blot assay (WB) of SLF-mAID-Ty with an expected MW of 143 kDa showing the regulation upon 24 h IAA addition. Actin, loading control. (D) IFA of double endogenously tagged strains showing colocalizations of SLF-mAID-HA with GC-Ty (and vice versa) and SLF-mAID-HA with UGO-Ty; (E) IFA performed on SLF-mAID-HA/GC-Ty strain, (F) SLF-mAID-HA/UGO-Ty, and (G) GC-mAID-HA/SLF-Ty with or without 24 h IAA addition. Actin, parasite cytoplasm; GAP45, parasite pellicle. Scale bar, 2 μm.

The protein encoded by TGGT1_208420 was previously reported as a putative GC-interacting protein based on the coimmunoprecipitation of GC after cross-linking followed by mass spectrometry analysis (10). TGGT1_208420 is predicted to encode 16 transmembrane-spanning domains (TMs) (35), and the region encompassing TMs 1 to 13 presents homology with a sodium neurotransmitter symporter superfamily-like domain (Fig. 1A) (36). TGGT1_208420 has recently been named SLF in a study reporting it as a signaling linking factor required for priming of parasite egress (37). *SLF* is predicted by a genome-wide fitness screen to be an essential gene due to its low fitness score (−2.3) (38). Unlike GC, SLF is not present in all members of the Apicomplexa phylum, being absent in *Cryptosporidia* and *Gregarines* (see Fig. S1D in the supplemental material). Based on a BLAST search, we found four genes (TGME49_208410, TGME49_285800, TGME49_314340, and TGME49_264870) showing sequence similarity to T. gondii SLF and that contain between 12 and 16 TM helices, according to HMMTOP prediction (Fig. S1D). To determine their localizations, we C-terminally tagged these SLF-like proteins with a 3-Ty tag construct (Fig. S1A). TGME49_208410, TGME49_314340, and TGME49_264870 all present a dotty intracellular localization (Fig. S1C), whereas the expression of TGME49_285800 was not detectable in tachyzoites by indirect immunofluorescence assay (IFA), although tagging was confirmed by genomic PCR (Fig. S1B). Transcriptome sequencing (RNA-seq) analysis indicates that TGME49_285800 transcripts are more abundant in the merozoite, although we could not rule out protein levels below the threshold of detection in the tachyzoite (ToxoDB). Notably, when searching homologues of SLF and SLF-like proteins in *Plasmodium*, PBANKA_0306700 (PF3D7_0209600) uniquely presents the highest sequence similarity to SLF, TGME49_208410, and TGME49_285800, as well as TGME49_264870. Notably, when searching homologues of PBANKA_0306700 in *T. gondii*, TGME49_285800 is the closest by sequence homology. PBANKA_0306700 presents multipass membrane domains (SNF-like domains), showing features of a sodium neurotransmitter symporter. The fitness scores of TGME49_208410, TGME49_285800, and TGME49_264870 suggest dispensability of these genes, which are possibly functionally redundant or perform dispensable tasks for the *in vitro* lytic cycle of the parasite. PBANKA_0306700 is instead predicted to be essential (39), and whether it participates in the GC complex in *Plasmodium* remains to be determined.

10.1128/mbio.01965-22.1 FIG S1 (A) Illustration of strategy for tagging proteins. A 3-Ty epitope tag and HXGPRT selection cassette were integrated in the C terminus of SLF-like proteins by CRISPR/Cas9-mediated homologous recombination. The red inverted triangle indicates the gRNA target sites. The primer pair (9955/3980) used to check the 3′ integration of the Ty tags for the TGME49_285800 gene locus (B) is indicated with the expected PCR product size (466 bp). (C) IFA showing localizations of endogenously tagged SLF-like proteins in *T. gondii*. GAP45, parasite pellicle; SAG1, parasite plasma membrane. Scale bar, 1 μm. (D) Overview of SLF and SLF-like proteins in Apicomplexa. The GeneID is the gene accession number provided by EuPathDB. PBANKA_0306700 is the homologue of TGME49_285800 in Plasmodium berghei. #TM, number of transmembrane domains predicted by HMMTOP. Localization was experimentally determined in this study. The essentiality was predicted by the genome-wide loss-of-function screen performed on T. gondii (Tgo). A negative score indicates parasite fitness loss for the knockout of the gene of interest. Conservation among the apicomplexans is based on a BLAST search (E value of <E−10). Green indicates the presence of genes. Download FIG S1, TIF file, 1.5 MB. Copyright © 2022 Ye et al. 2022 Ye et al. https://creativecommons.org/licenses/by/4.0/ This content is distributed under the terms of the Creative Commons Attribution 4.0 International license.

To investigate the function of SLF, we destabilized the protein via the auxin degron system (40). The *SLF* endogenous locus was fused with a mini-auxin degron-Ty tag (mAID-Ty) coding sequence in the parental RH/Tir1 (Tir1) strain, and the clonal parasites were confirmed by genomic PCR (Fig. S2A and B). The resulting tagged SLF-mAID protein localized to the apical tip and basal pole of the parasites by IFA, as previously reported for the components of the GC complex (Fig. 1B). SLF-mAID-Ty migrated at the expected size of 143 kDa and was tightly regulated upon treatment of SLF-mAID-Ty parasites with indole-3-acetic acid (IAA) for 24 h (Fig. 1B and C), but it was still detectable after 6 h of treatment (Fig. S2C). Degradation of SLF-mAID-Ty by the proteasome confirms the predicted topology, with the C terminus of the protein facing the cytosol of the parasite.

10.1128/mbio.01965-22.2 FIG S2 (A) Illustration of the auxin-inducible degron (AID) system to generate a conditional knockdown of TgSLF. The C terminus of TgSLF was fused with a mini-AID, a 3-Ty epitope tag, and the HXGPRT cassette by CRISPR/Cas9-mediated homologous recombination. The red inverted triangle indicates the gRNA target site. The pair of primers indicated (9916/9824) makes a 486-bp PCR product to check the 3′ integration of the mini-AID to the SLF gene locus. (B) Diagnostic PCR for the construction of the SLF-mAID-Ty strain using primers 9916/9824. (C) Western blot performed on the SLF-mAID-Ty strain to check the regulation of SLF by the AID system. Parasites were treated with 500 μM IAA for 0, 6, or 24 h. Actin, loading control. (D) Representative image of conoid extrusion assay performed on the SLF-mAID-Ty strain. The preconoidal rings and apical cap of parasites were found to be methylated and were detectable by IFA staining with pan-*N*-ε-trimethyl lysine antibody. Actin, parasite cytoplasm. Arrowheads point out the extruded preconoidal rings. Scale bar, 1 μm. (E) Quantification of extruded conoid in different strains treated with or without BIPPO based on IFA staining with anti-actin and anti-methylated lysine antibodies (*n* = 3). ***, *P* < 0.001, and **, *P* < 0.01, by two-tailed Student’s *t* test. C1, compound 1 (a PKG inhibitor) (71); CD, cytochalasin D, an actin polymerization inhibitor (44). (F) Quantification of F-actin basal accumulation in different strains without BIPPO treatment. Download FIG S2, TIF file, 2.1 MB. Copyright © 2022 Ye et al. 2022 Ye et al. https://creativecommons.org/licenses/by/4.0/ This content is distributed under the terms of the Creative Commons Attribution 4.0 International license.

To confirm that it belongs to the GC complex, SLF was C-terminally tagged with 2 Ty epitopes at the endogenous locus in the GC-mAID-HA (hemagglutinin) strain. Reciprocally, GC and UGO were C-terminally tagged with 2 Ty epitopes at the endogenous locus in the SLF-mAID-HA strain. SLF-mAID-HA colocalized with GC and UGO (Fig. 1D), and its deletion perturbed the localization of the partner proteins (Fig. 1E and F). Conversely, in the absence of GC, SLF no longer accumulated to the apical tip and the residual body of intracellular parasites (Fig. 1G). Taken together these results suggest that SLF belongs to GC complex and participates in its assembly and trafficking to the parasite plasma membrane.

### SLF depletion phenocopies GC depletion.

**FIG 2:**
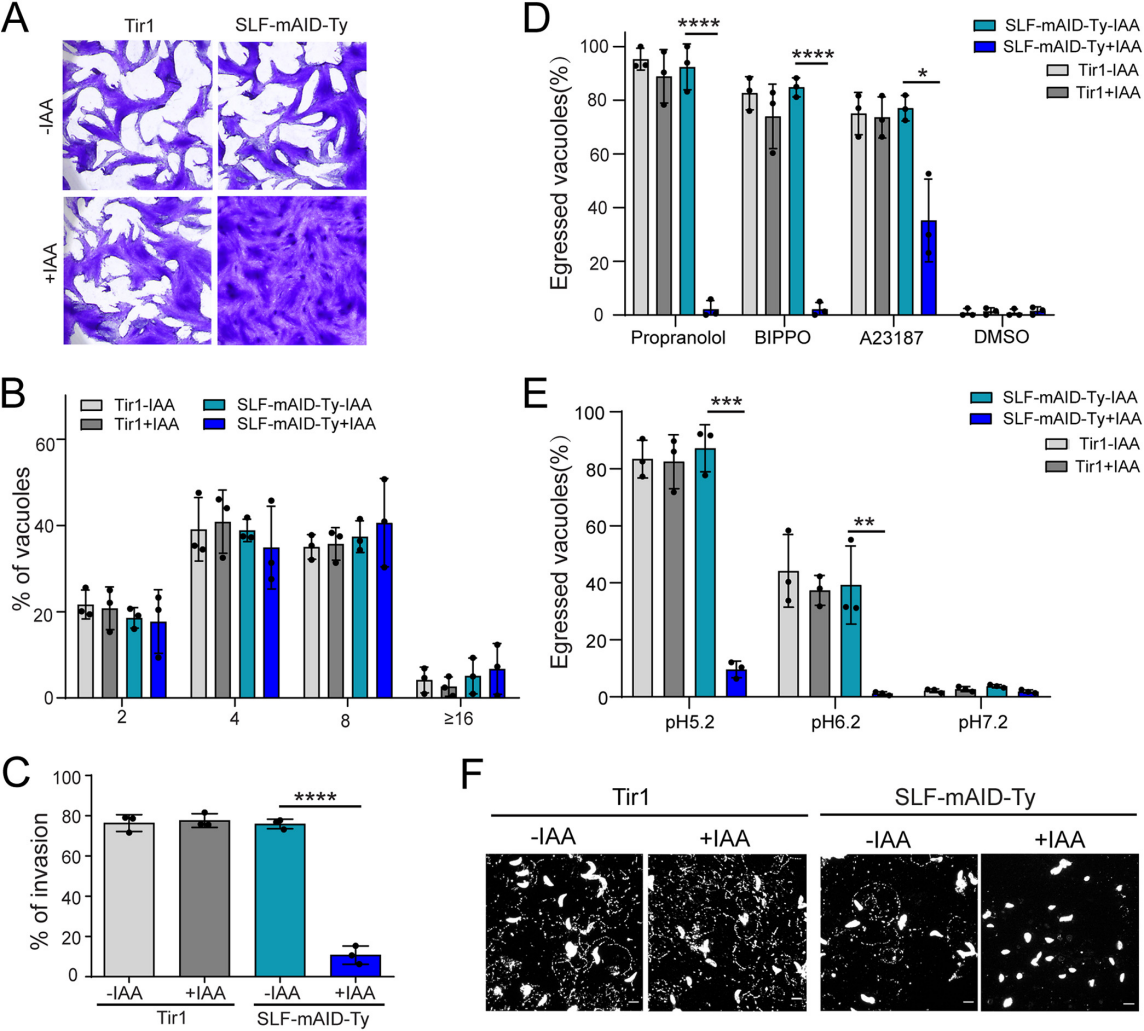
SLF is essential for tachyzoite invasion, gliding motility, and egress. (A) Plaque assay of SLF-mAID-Ty and Tir1 parasites. With 7 days of IAA treatment, the SLF mutant is not capable of forming plaques (*n* = 5). (B) Intracellular growth assay of SLF-mAID-Ty upon 24 h growth with or without IAA. The numbers of PVs containing 2, 4, 8, or 16 parasites were counted by IFA (*n* = 3). (C) Red/green invasion assay to check the invasion capacity of SLF-mAID-Ty with or without 24 h IAA pretreatment. Results are presented as the percentages of invaded parasites for each group (*n* = 3). ****, *P* < 0.0001 by two-tailed Student’s *t* test. (D) Induced egress assay of SLF-mAID-Ty upon propranolol, BIPPO, A23187, or DMSO treatment. Results are presented as the percentages of broken PVs (*n* = 3). ****, *P* < 0.0001, and *, *P* < 0.05, by two-tailed Student’s *t* test. (E) Egress assay exhibiting the responses of intracellular parasites to different pHs. Results are presented as percentages of broken PVs (*n* = 3). ***, *P* < 0.001, and **, *P* < 0.01, by two-tailed Student’s *t* test. (F) Representative images of gliding/trail assay performed on SLF-mAID-Ty and Tir1 strains (*n* = 3). Gliding trails were visualized by SAG1 staining. Scale bar, 5 μm.

The phenotyping of the SLF-mAID-Ty strain revealed that SLF-depleted parasites were severely affected in the lytic cycle, being unable to form visible plaques on human foreskin fibroblast (HFF) monolayers upon addition of IAA for 7 days (Fig. 2A). However, the intracellular growth assay clearly indicated that SLF depleted parasite replication normally (Fig. 2B). In contrast, SLF deletion caused a severe impairment of parasite invasion (Fig. 2C). The parasite’s ability to egress from infected cells was assessed using several triggers to explore the role of SLF at different steps of the cGMP-PKG signaling pathway. BIPPO inhibits PDEs, leading to cGMP accumulation (41), propranolol targets phosphatidic acid phosphatase (PAP), causing PA upregulation (42), and calcium ionophore A23187 raises the intracellular calcium concentration (43). When induced by BIPPO, SLF-depleted parasites presented a complete block in egress, suggesting loss of cGMP production (Fig. 2D). Parasite egress induced by A23187 was only partially impaired, indicating that increased calcium levels could partially rescue SLF loss, as previously shown for GC depletion (10) (Fig. 2D). Based on a previous model, accumulated PA induced by propranolol serves as a sensor and elicits microneme secretion (19), which is necessary but not sufficient for parasite egress (20). Propranolol failed to induce egress of SLF-depleted parasites, indicating that GC activity leading to Ca^2+^ signaling is critical for egress (Fig. 2D). Natural egress of tachyzoites is accompanied by a drop of vacuole pH, which could lead to GC activation (10, 11). Incubation of parasites at pH 5.2 induces 80% of parasite egress, which was blocked by the depletion of SLF, suggesting a possible role of SLF in GC activation in response to acidic pH, among other stimuli (Fig. 2E). Invasion and egress rely on parasite motility (4), which can be assessed by detecting the trails of the tachyzoite surface membrane protein 1 (SAG1) deposited by moving parasites on a coated surface (44). A marked reduction of trail formation uncovered the abrogated gliding capacity of SLF-depleted parasites after IAA treatment (Fig. 2F).

**FIG 3:**
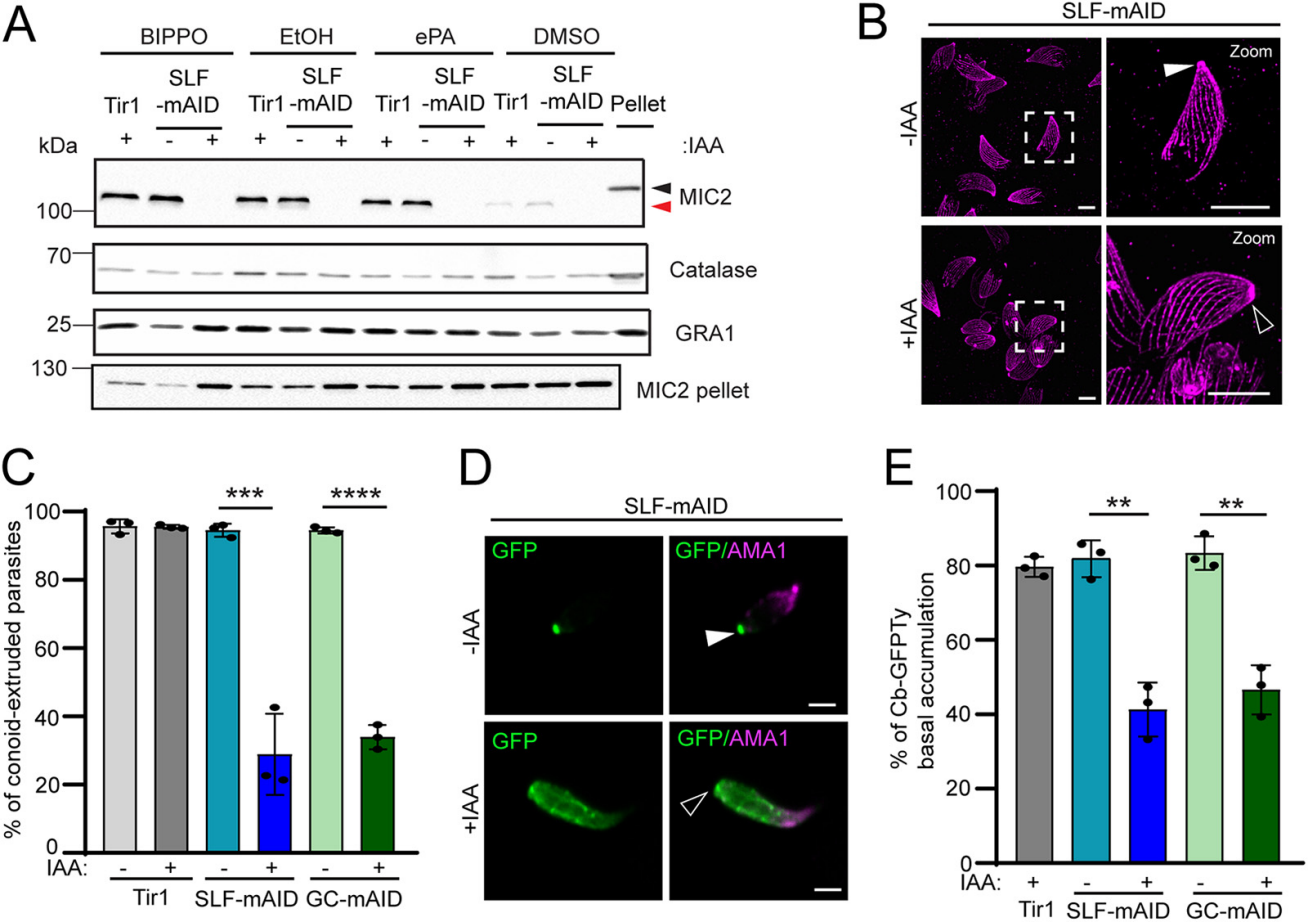
SLF regulates microneme secretion, conoid extrusion, and F-actin basal accumulation. (A) Microneme secretion assay showing the defect of microneme exocytosis caused by SLF depletion. BIPPO, ethanol, and ePA are triggers for parasite microneme secretion. DMSO is a negative control. Red and black triangles indicate processed MIC2 in excreted and secreted supernatant and unprocessed MIC2 in the pellet fraction, respectively. Catalase, nonsecreted protein as a marker for parasite lysis; GRA1, constitutive secreted protein as an internal reference. (B) Representative images of conoid extrusion assay performed on the SLF-mAID-Ty strain. U-ExM was performed to clearly view intraconoidal microtubules when parasites were stained with anti-α-tubulin and anti-β-tubulin antibodies. Enlarged images of one conoid extruded parasite and one conoid nonextruded parasite are framed in white dashed boxes on the right. Solid arrowhead, extruded conoid; open arrowhead, nonextruded conoid. Scale bar, 5 μm. (C) Quantification of conoid extruded parasites. (*n* = 3). ****, *P* < 0.0001, and ***, *P* < 0.001, by two-tailed Student’s *t* test. (D) Representative images of F-actin staining. GFP indicates F-actin. AMA1, a marker of parasite apical tip. Solid arrowhead, F-actin basal accumulation. Open arrowhead, absence of F-actin basal accumulation. Scale bar, 2 μm. (E) Quantification of F-actin basal accumulated parasites (*n* = 3). **, *P* < 0.01 by two-tailed Student’s *t* test.

Given that SLF is an essential component of the GC complex, we anticipated an impairment in microneme secretion, conoid extrusion and apico-basal flux of F-actin upon SLF deletion. Microneme exocytosis of SLF-depleted parasites was scrutinized using extracellular PA (ePA), BIPPO, and ethanol as triggers. Ethanol leads to elevation of Ca^2+^—probably by activating PI-PLC (45). Processed MIC2, indicative of microneme secretion and protein shedding from the parasite plasma membrane, is quantified in the excretory-secretory antigen (ESA) fraction. The absence of SLF abolished microneme exocytosis under all three conditions (Fig. 3A). The conoid is a small dynamic organelle composed of tubulin-rich fibers (46). A quantitative conoid extrusion assay induced by BIPPO on extracellular parasites and visualized with tubulin antibodies by ultrastructure expansion microscopy (U-ExM) was applied to SLF-mAID-Ty (47). Depletion of SLF as well as GC resulted in a dramatic decrease of conoid extrusion (Fig. 3B and C). The defect of conoid extrusion resulting from SLF or GC depletion was confirmed based on the staining of methylated preconoidal rings that top the conoid (Fig. S2D and E) (8). To monitor the apico-basal flux of F-actin, SLF-mAID-Ty, GC-mAID-HA, and Tir1 were transiently transfected with pT8-Cb-GFP expressing green fluorescent protein (GFP)-Ty fused to anti-actin chromobodies (GFP-Ty-Cb) (Fig. 3D) (5). While around 80% of BIPPO-stimulated parental parasites showed accumulation of F-actin at the basal pole, parasites depleted in SLF or GC exhibited a partial phenotype, with around 45% of parasites accumulating F-actin at the posterior (Fig. 3E and Fig. S2F). Taken together, tachyzoites depleted in SLF are impaired in microneme exocytosis, conoid extrusion, and motility and hence unable to invade and egress, supporting SLF as a key signaling factor for tachyzoite dissemination.

### The GC complex contributes to the onset of chronic infection.

The GC complex receives and transduces signals for tachyzoite egress, motility, and invasion, contributing to tachyzoite dissemination. According to transcriptional profiling of T. gondii from ToxoDB, SLF, GC, UGO, and CDC50.1 are also expressed during chronic infection (48). Additionally, bradyzoite gliding motility was recently shown to depend on calcium signaling (33), suggesting that GC could operate to control bradyzoites’ invasion, motility, or egress. To determine if bradyzoites have the capacity to actively egress and reinvade host cells, contributing to cyst burden, we assessed the role of SLF and GC in bradyzoites using the mouse model of infection.

**FIG 4:**
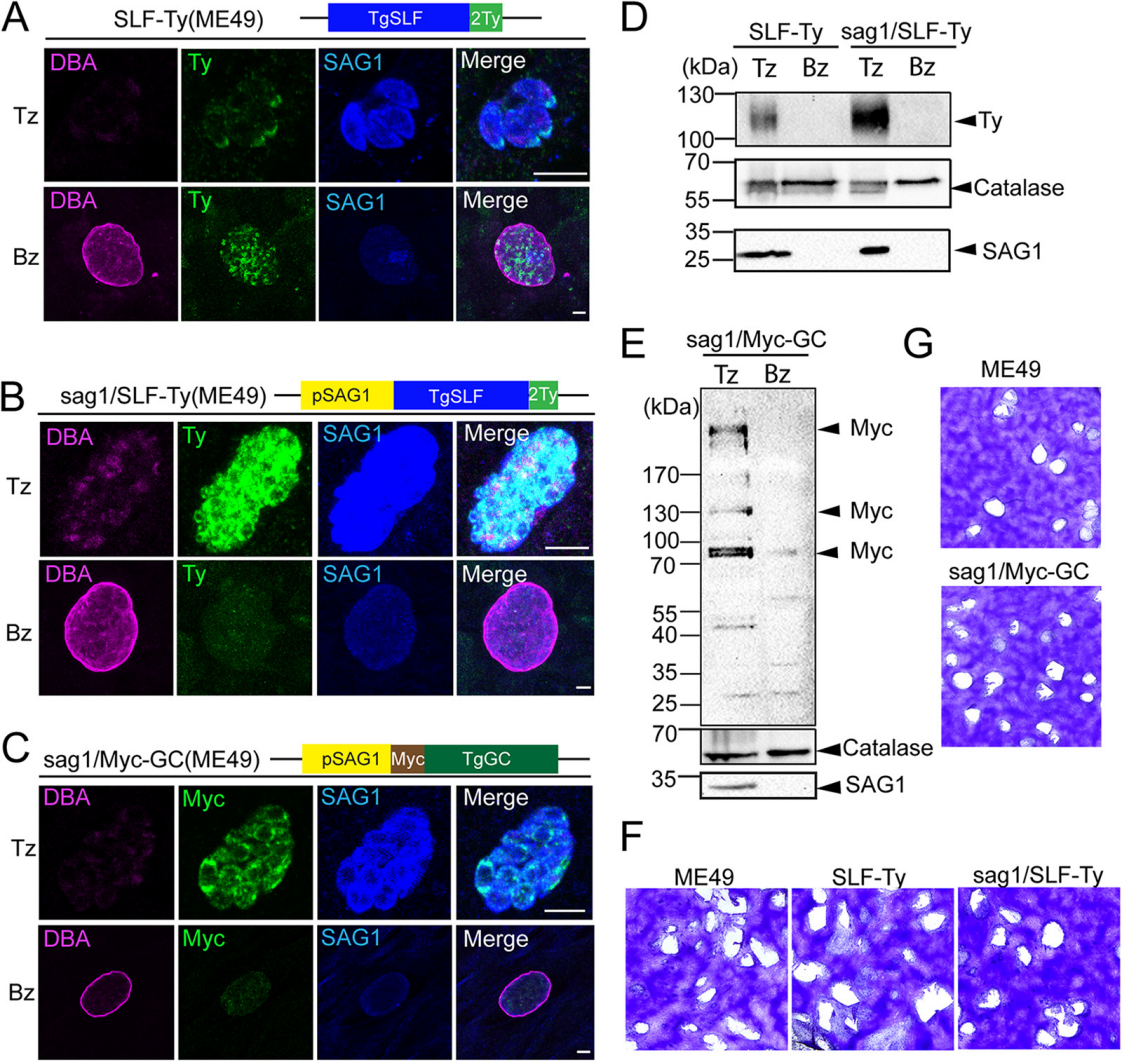
Promoter swap allows stage-specific expression of key GC complex factors. (A and B) IFA showing the expression and localization of SLF before and after promoter swap in tachyzoites and 1-week differentiated bradyzoites. DBA, Dolichos biflorus agglutinin, a marker of cyst wall; SAG1, an indicator of tachyzoite stage; Tz, tachyzoite; Bz, bradyzoite. Scale bar, 5 μm. (C) IFA showing the expression and localization of GC in tachyzoites and 1-week differentiated bradyzoites of sag1/Myc-GC. Scale bar, 5 μm. (D) WB performed on SLF-Ty and sag1/SLF-Ty showing expression levels of SLF in tachyzoites and 1-week differentiated bradyzoites. SAG1, a marker for tachyzoites; Catalase, loading control. (E) Comparison of GC expression levels of sag1/Myc-GC between tachyzoite and bradyzoite stages by WB analysis. (F) plaque assay to check the fitness of SLF-Ty and sag1/SLF-Ty tachyzoites *in vitro*; (G) plaque assay of sag1/Myc-GC tachyzoites.

Given the crucial contribution of SLF and GC for several steps of the tachyzoite lytic cycle, studying the importance of these proteins in bradyzoites during *in vivo* infection requires the use of a stage-specific knockdown system. Current inducible systems present two major issues for gene study in bradyzoites during chronic infection in the animal model. The reduced permeability of the blood-brain barrier and of the cyst wall could limit the access of small-molecule inducers to bradyzoites colonizing the brain (49). Moreover, the uncertain dynamics of tachyzoite-bradyzoite conversion complicates the timing and dosing of inducers. To overcome this limitation, we opted for a promoter swap strategy, placing the gene of interest under the transcriptional control of the tachyzoite-specific *SAG1* promoter, which is silenced upon conversion to bradyzoites (50, 51). The virulent type I strain RH/Tir1 causes lethal infection of mice during the acute phase, making it not suitable for chronic infection studies. In contrast, the type II strain ME49 presents attenuated virulence with increased ability to form cysts (52). The promoter swap strains of SLF and GC were generated in the cystogenic ME49 strain. Of relevance, differentiation from tachyzoites to bradyzoites is achievable *in vitro*, using alkaline medium for parasite culture (53). Antibodies against SAG1 and Dolichos biflorus agglutinin (DBA) lectin, the latter binding to cyst wall carbohydrates, were used in the IFA to monitor efficiency of stage conversion (54, 55). SLF was first tagged with Ty epitope in the endogenous locus. Tachyzoites, as well as 7-day-induced *in vitro* bradyzoites, were observed presenting the same localization of SLF as that in Tir1 strain (Fig. 4A). Next, the endogenous promoters of SLF and GC were swapped with the *SAG1* promoter to generate sag1/SLF-Ty and sag1/Myc-GC, respectively. The resulting clonal strains were confirmed by genomic PCR (Fig. S3A to D). Promoter swap resulted in overexpression of SLF, and likely of GC, in tachyzoites (Fig. 4B and C). Upon conversion of these strains to bradyzoites by induction with alkaline media for 7 days, SLF and GC signals disappeared almost completely by IFA (Fig. 4B and C). Endogenous SLF-Ty in tachyzoites migrates at the expected size of 120 kDa by western blotting of tachyzoites, but it was not detectable in *in vitro* bradyzoites. After the promoter swap, SLF showed a strong increase in level of expression in tachyzoites and remained undetectable in bradyzoites (Fig. 4D). As previously described, GC displayed multiple bands by western blotting, mainly represented by the functional full length of GC (477 kDa) and two breakdown products (~130 and 80 kDa) (13, 14). Full-length and small breakdown products were dramatically decreased upon sag1/Myc-GC conversion to bradyzoites (Fig. 4E). It is noteworthy that overexpression of SLF and GC caused by promoter swap did not alter parasite fitness based on the plaque assay and intracellular growth assay (Fig. 4F and G and Fig. S4A).

10.1128/mbio.01965-22.3 FIG S3 (A) Cartoon representing the promoter swap strategy applied for SLF. The C terminus of TgSLF was fused with a 2-Ty epitope tag and DHFR cassette by single-guide RNA (gRNA) based on CRISPR/Cas9-mediated homologous recombination in ME49 parasites. Subsequently, the original SLF promoter region was replaced with SAG1 promoter (pSAG1) sequence holding 40-bp SLF homology arms by 2 gRNAs directed by a CRISPR/Cas9 genome editing approach in the SLF-tagged strain (SLF-Ty). Flags represent predicted promoter start regions. Red inverted triangles indicate gRNA target sites. Positions of primers used for diagnostic PCR are marked. (B) Diagnostic PCRs for the SLF promoter swap strain. Pairs of primers (9515/10213 and 1935/10214) were used to check the 5′ and 3′ integration of SAG1 promoter sequence to the SLF 5′ untranslated region (UTR). A pair of primers (10213/10215) was used to examine the excision of the SLF original promoter region. Predicted PCR product sizes are indicated. (C) Schematic illustrating the construction of sag1/Myc-GC promoter swap mutant by CRISPR/Cas9-assisted homologous gene replacement. ME49 parasites were transfected with a Cas9-expressing plasmid and 2 gRNAs to generate double-strand cuts in the predicted GC promoter region. DNA repair was achieved by cotransfection with a homology template carrying the SAG1 promoter, a Myc tag, and 40 bp of homology to the locus on each side. Flags represent predicted promoter start regions. Red inverted triangles indicate gRNA target sites. Positions of primers used for diagnostic PCR are marked. (D) PCR identification of sag1/Myc-GC construction. The proper integration of SAG1 promoter in sag1/Myc-GC parasites was checked by PCR using primers (9515/10056 and 1935/10057). A pair of primers (10056/10058) was used to examine the loss of GC original promoter region in sag1/Myc-GC. Predicted PCR product sizes are indicated. (E) PCR identification for sag1/SLF-Ty cysts. Primers are positioned in panel A, and predicted PCR product sizes are indicated. *, tissue cyst. (F) PCR identification for sag1/Myc-GC cysts. Primers are positioned in panel C, and predicted PCR product sizes are indicated. *, tissue cyst. Download FIG S3, TIF file, 1.5 MB. Copyright © 2022 Ye et al. 2022 Ye et al. https://creativecommons.org/licenses/by/4.0/ This content is distributed under the terms of the Creative Commons Attribution 4.0 International license.

10.1128/mbio.01965-22.4 FIG S4 (A) Intracellular growth assay of sag1/SLF-Ty and sag1/Myc-GC. The numbers of PVs containing 2, 4, 8, or 16 parasites were counted. At least 200 PVs were analyzed for each condition (*n* = 3). (B) Plaque assay of ME49, sag1/SLF-Ty, and sag1/Myc-GC tachyzoites used for mouse intraperitoneal injection, representative from one experiment. (C) Quantification of plaque numbers from two independent experiments in panel B. Open and solid symbols represent plaque numbers from the first and second experiments, respectively. (D) Plaque assay of ME49, sag1/SLF-Ty and sag1/Myc-GC cysts orally given to mice, representative from one experiment; (E) quantification of plaque numbers from two independent experiments in panel D. Open and solid symbols represent plaque numbers from the first and second experiments, respectively. (F) Western blot checking seroconversion of mice against *T. gondii*. HFF cells inoculated with (+ label) or without (− label) ME49 tachyzoites were loaded on gel. Two western blot membranes show seroconversion of mice from two animal experiments. The numbers 1 to 6 represent the individual mice from each group. *, tissue cyst. Download FIG S4, TIF file, 1.4 MB. Copyright © 2022 Ye et al. 2022 Ye et al. https://creativecommons.org/licenses/by/4.0/ This content is distributed under the terms of the Creative Commons Attribution 4.0 International license.

**FIG 5:**
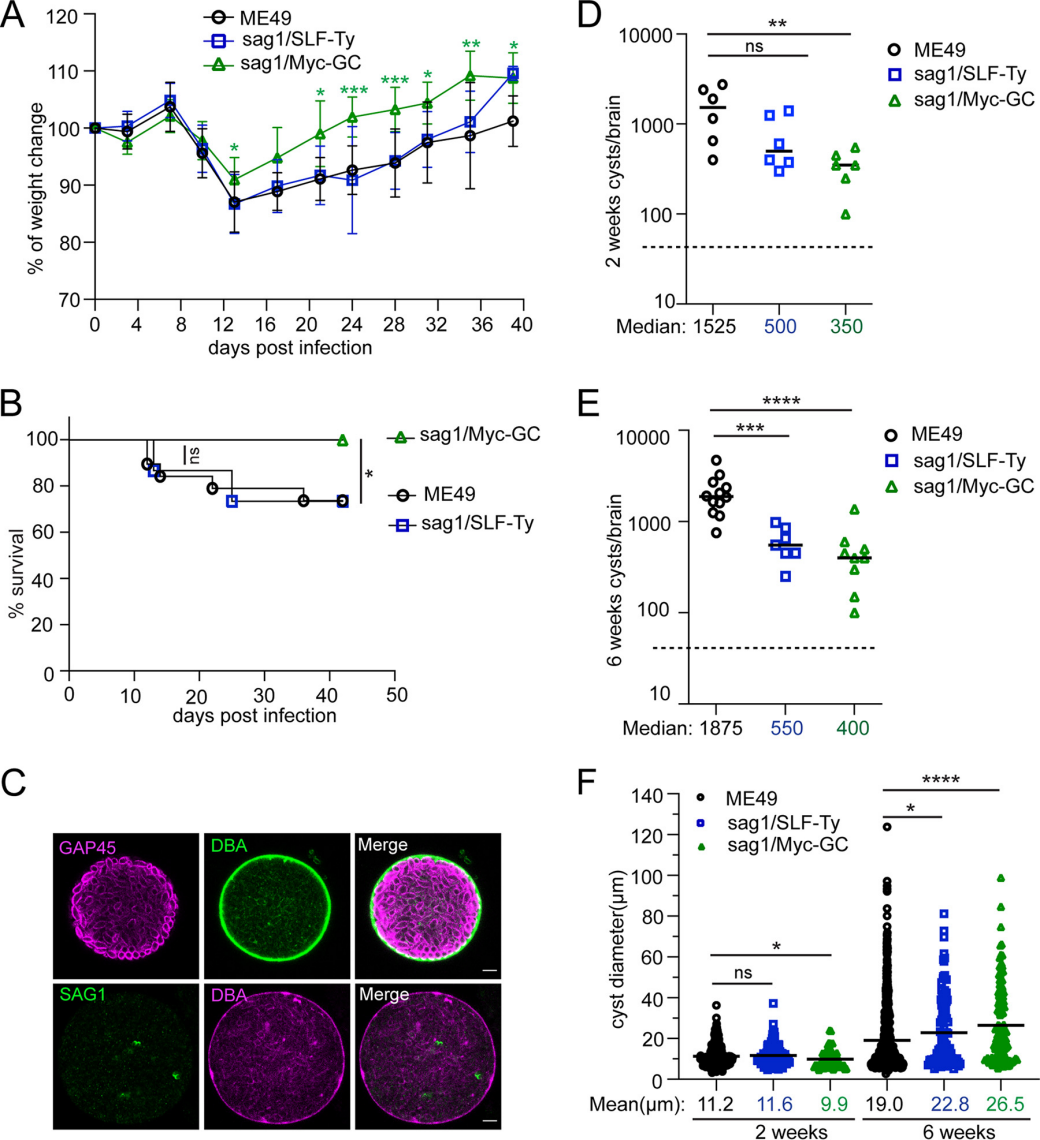
GC complex contributes to the onset of chronic infection. (A) Body weight change of mice. Mice were intraperitoneally infected with sag1/SLF-Ty (*n* = 15), sag1/Myc-GC (*n* = 15), or ME49 (*n* = 19) and weighed every 3 to 4 days from the day of infection. Body weight change is reported as percentage compared to day 0 (uninfected). Data were pooled from two independent experiments (for sag1/Myc-GC compared to the ME49 group, ***, *P* < 0.001; **, *P* < 0.01, and *, *P* < 0.05, by two-tailed Student’s *t* test). (B) Survival curve of mice intraperitoneally challenged with low dose of ME49 (*n* = 19; 14 survived), sag1/SLF-Ty (*n* = 15; 11 survived), and sag1/Myc-GC (*n* = 15; 15 survived) tachyzoites (from two independent experiments). Mice euthanized for cyst counting are excluded from the survival curve. *, *P* < 0.05, and ns, not significant, by logrank Mantel-Cox test. (C) Representative immunostaining images of *ex vivo* cysts. GAP45, parasite pellicle. DBA, cyst wall. SAG1, tachyzoite plasma membrane. Scale bar, 5 μm. (D and E) Cyst burdens in mouse brains infected with ME49, sag1/SLF-Ty, and sag1/Myc-GC tachyzoites for 2 weeks (D) or 6 weeks (E) (from two independent experiments). An individual dot represents a cyst count from one mouse. Medians of cyst numbers are highlighted by short black lines. The dotted line indicates the limit of detection (50 cysts/brain). A log_10_ scale is used for the *y* axis. ****, *P* < 0.0001; ***, *P* < 0.001; **, *P* < 0.01, and ns, not significant, by two-tailed Mann-Whitney test. (F) Size distribution of tissue cysts harvested from 2 weeks and 6 weeks postinfection (from two independent experiments). One dot represents the size of one cyst. Mean cyst sizes are highlighted by short black lines. ****, *P* < 0.0001; *, *P* < 0.05, and ns, not significant, by two-tailed Mann-Whitney test.

To develop chronic toxoplasmosis, mice were intraperitoneally injected with a low dose of sag1/SLF-Ty, sag1/Myc-GC, or ME49 tachyzoites. A total of 150 counted tachyzoites of each strain produced an average 36 to 45 plaques on HFF monolayers, confirming that equal amounts of viable parasites were inoculated for each mouse (Fig. S4B and C). Animal health status was monitored daily, and the body weight of the mice was traced regularly. As expected, mice from each group lost weight during the second week of inoculation and afterwards gradually gained the weight back, which indicates that two promoter swap tachyzoites have the capacity to develop the acute phase of the disease (Fig. 5A). Accordingly, we presumed that tachyzoites of the two mutants were not defective in passing the blood-central nervous system (CNS) barrier to colonize the brain parenchyma. Over the course of the experiment, mice were humanely euthanized once they presented severe toxoplasmosis symptoms (difficulty moving, lack of response to stimuli, and/or steady weight loss). Virulence of sag1/SLF-Ty parasites resulted in 26.6% of infected mice being sacrificed, showing a virulence comparable to that of ME49 (26.3%). In contrast, no mouse succumbed to sag1/Myc-GC infection (Fig. 5B).

It has been shown that tissue cyst burden caused by the ME49 strain in mouse brain remains stable between weeks 3 and 8 postinfection (29). We investigated whether an improvement in cyst number in mouse brain is detectable between week 2 (early infection) and week 6 (advanced infection) postinfection. To investigate the contribution of SLF and GC to the establishment of chronic infection, mice were sacrificed to determine cyst burdens in the brain at these two time points. Mouse brains were homogenized by syringe passage to allow release of tissue cysts. Afterwards, DBA and GAP45 staining was performed to visualize tissue cysts and parasites to help in the quantification of cyst numbers, size, and cyst and parasite morphology (Fig. 5C). Compared to the ME49 parental strains, both transgenic parasites led to reductions of cyst numbers in 2-week-infected animals, although the decreases in cyst numbers by sag1/SLF-Ty parasites were not statistically significant (Fig. 5D). Consistent with the result from week 2 postinfection, sag1/SLF-Ty and sag1/Myc-GC parasites formed significantly fewer cysts in mouse brains than their parental line at week 6 postinfection (Fig. 5E). Of note, the tissue cyst burden of all parasite lines remained stable rather than increasing over time, implying that expansion of cyst number likely happens before 2 weeks postinfection, and since then, bradyzoite cysts rarely spread. Collectively, we ascribe the spread of the cyst population during the onset of chronic infection to both tachyzoite colonization and bradyzoite dissemination, which appear to be compromised by the absence of SLF or GC.

In terms of cyst size, minor differences between groups were observed at 2 weeks postinfection (Fig. 5F). Cyst sizes of all parasite strains at 6 weeks postinfection were increased compared to these at 2 weeks postinfection, with large variation in sizes within groups (Fig. 5F), explained by slow-growing bradyzoites and cyst wall expansion (29). Six-week cysts formed by knockdown strains were significantly larger than 6-week cysts of their parental line (Fig. 5F). We hypothesize that with sustained replication rates, reduced cyst rupture could lead to accumulation of bigger cysts. As expected, no obvious cyst morphology defect in knockdown strains was detected by IFA with DBA and GAP45 markers (Fig. 5C).

Altogether, these data suggest that GC and SLF significantly contribute to the onset of chronic infection. For long-term chronic infection, GC and SLF might be involved in cyst rupture and turnover, although this does not lead to an increase in cyst numbers.

### SLF is essential for establishment of natural bradyzoite infection.

During natural infection, upon ingestion of tissue cysts, the gastric acid and pepsin dissolve the cyst wall and release bradyzoites into the intestinal ileum. Bradyzoites invade intestinal epithelium cells, switching to tachyzoites, leading to an acute infection and chronic infection sequentially. Conversely, tachyzoites lacking cyst wall protection will be immediately damaged by the gastric acid (39). We expect that depletion of SLF or GC impairs invasion, motility, and egress of *in vivo* bradyzoites, leading to bradyzoite digestion, as observed for the tachyzoite.

**FIG 6:**
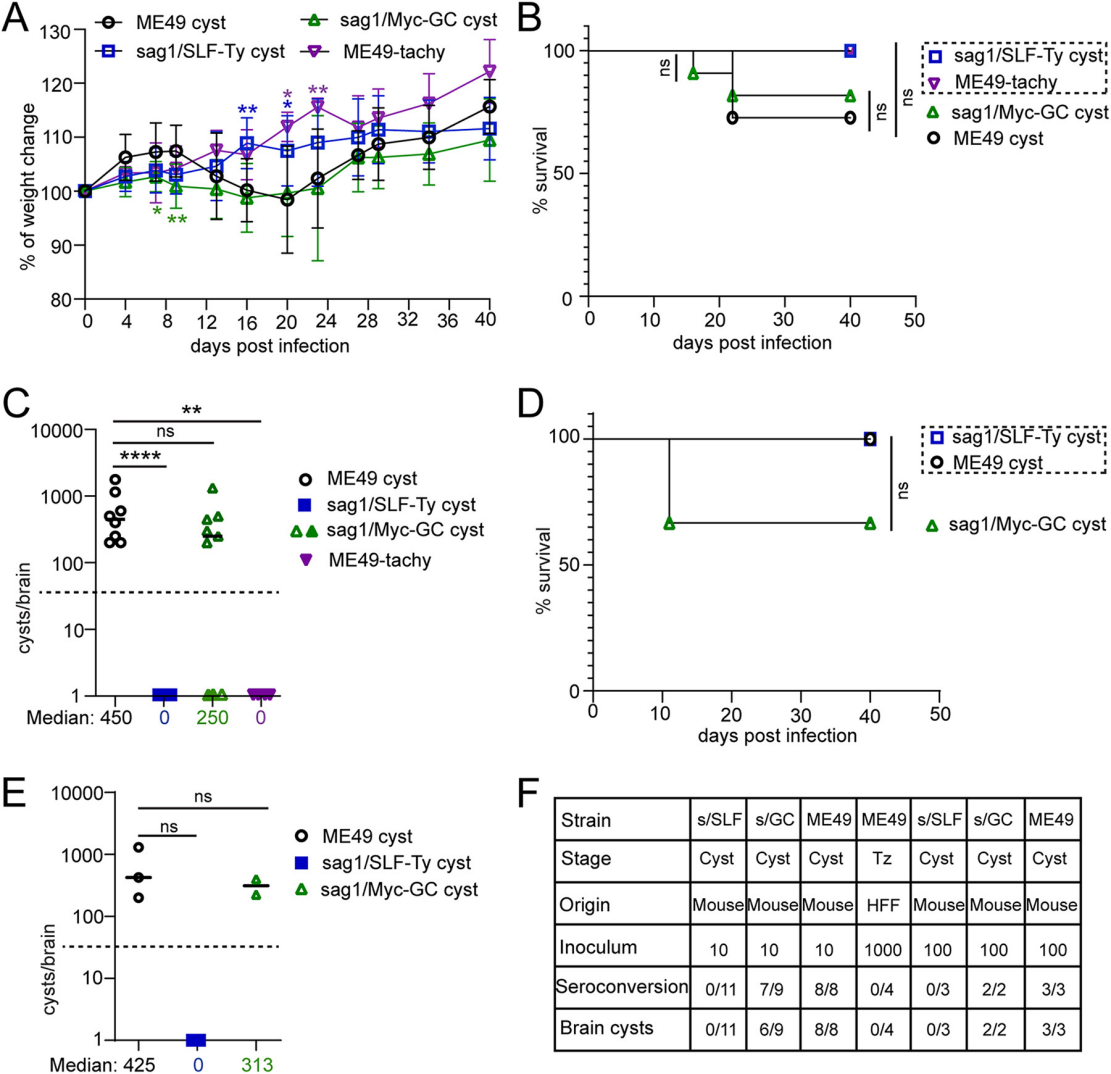
SLF is required for establishment of natural bradyzoite infection. (A) Body weight change of mice orally infected by gavage with *ex vivo* cysts of ME49 (*n* = 11), sag1/SLF-Ty (*n* = 11), or sag1/Myc-GC (*n* = 11) or with ME49 tachyzoites (*n* = 4). Mice were weighed every 3 to 4 days from the day of infection until day 40 postinfection. Body weight change is reported as percentage compared to day 0 (uninfected). Data were pooled from two independent experiments. **, *P* < 0.01, and *, *P* < 0.05, by two-tailed Student’s *t* test. (B) Survival curve of mice that orally received 10 *ex vivo* cysts of ME49 (*n* = 11; 8 survived), sag1/SLF-Ty (*n* = 11; 11 survived), or sag1/Myc-GC (*n* = 11; 9 survived) or 1000 ME49 tachyzoites (*n* = 4; 4 survived). Data were collected from two independent experiments. ns, not significant by logrank Mantel-Cox test. (C) Cyst numbers of mice following oral gavage with different strains for 6 weeks. Individual symbol represents cyst count from one mouse. Solid symbols present counts from seronegative mice. The dotted line indicates the limit of detection (50 cysts/brain). The median cyst number is presented. The graph is on a log scale. Data were pooled from two independent experiments. ****, *P* < 0.0001; **, *P* < 0.01, and ns, not significant, by two-tailed Mann-Whitney test. (D) Survival curve of mice orally infected with 100 tissue cysts of ME49 (*n* = 3; 3 survived), sag1/SLF-Ty (*n* = 3; 3 survived), and sag1/Myc-GC (*n* = 3; 2 survived). ns, not significant by logrank Mantel-Cox test. (E) Cyst count from mouse brains infected with 100 tissue cysts of three strains for 6 weeks. One symbol represents a count from one mouse. Solid symbols present counts from seronegative mice. ns, not significant by two-tailed Mann-Whitney test. (F) Summary of oral infection experiments. Seroconversion and brain cysts were checked for mice that survived until the endpoints.

To verify our hypothesis, a naive batch of mice were challenged by oral gavage with *ex vivo* cysts collected from chronically infected mice (6 weeks postinfection), mimicking the natural *Toxoplasma* infection. Mouse infection with ME49 tachyzoites was performed as a critical control. Before oral gavage, cyst strains were verified by PCR (Fig. S3E and F). An inoculum of 10 counted cysts from each strain produced an average of 26 to 29 plaques on HFF cells, indicative that collected cysts were infectious to *in vitro* culturing HFFs, and comparable amounts of these cysts were given to each mouse (Fig. S4D and E). Cysts produced by sag1/Myc-GC parasites, like ME49 cysts, elicited weight loss of infected mice between days 8 and 20 postinoculation (Fig. 6A). Contrarily, throughout the period, mice infected with sag1/SLF-Ty cysts and tachyzoites showed no symptoms of toxoplasmosis and gained weight (Fig. 6A). While no mouse succumbed to the oral infection with sag1/SLF-Ty or ME49 tachyzoites, sag1/Myc-GC cysts exhibited virulence comparable to that of ME49 cysts, resulting in ~20% of animals to be sacrificed (Fig. 6B). Cyst burden in the brain was determined 6 weeks after oral infection. Inoculation of sag1/Myc-GC cysts produced a reduced number of cysts in mouse brains compared to inoculation of the same amount of ME49 cysts, although this difference was not statistically significant. Remarkably, no tissue cyst was observed in the infection with sag1/SLF-Ty cysts. As expected, mice infected by oral gavage with ME49 tachyzoites presented no cysts (Fig. 6C). To determine the *Toxoplasma* infection of cyst-free mice, seroconversion of mice was checked. Three mice from the sag1/Myc-GC group presented no cysts, two of which were not seroconverted to T. gondii. All mice orally infected with sag1/SLF-Ty cysts as well as ME49 tachyzoites lacked seroconversion, demonstrating that these strains were not infectious when fed to mice (Fig. 6F and Fig. S4F).

To further test the defect in oral gavage infection by sag1/SLF-Ty as well as sag1/Myc-GC cysts, we set up a high-dose infection group with 100 cysts per mouse. One out of three mice that received 100 sag1/Myc-GC cysts needed to be culled during acute infection, while no mice succumbed to oral infection with sag1/SLF-Ty as well as ME49 cysts (Fig. 6D). Moreover, inoculation of 100 GC knockdown cysts produced fewer but not statistically significantly brain tissue cysts than inoculation of 100 ME49 cysts (Fig. 6E). Concordantly, all mice inoculated with sag1/SLF-Ty cysts failed to develop antibodies against T. gondii, and no cysts were detected in their brains, further confirming that cyst bradyzoites depleted in SLF are not infectious in the host gut (Fig. 6E and F and Fig. S4F).

Taken together, oral gavage of sag1/SLF-Ty cysts induced neither serum conversion of mice against T. gondii nor tissue cyst formation in mouse brain, providing clear evidence that SLF is a key factor for the establishment of natural bradyzoite infection.

## DISCUSSION

As an intracellular parasite, T. gondii has developed strategies to cope with various environments. PA, acting as a molecular clock, triggers tachyzoite natural egress (10). Parasites escape from unhealthy host cells at any time point of their replication by sensing external signals, such as changes in pH or ionic composition. These signals are integrated into a GC complex that has three known factors: GC, UGO, and CDC50.1. As a response, cGMP produced by GC serves as the key mediator, activating the signaling cascade, leading to the parasites’ egress (20). Here, we characterized SLF as an additional and essential component of the GC complex. SLF depletion leads to a severe loss of parasite fitness, concordant with the low fitness score of the genome-wide CRISPR loss-of-function study (38). No phenotypic discrepancy was found among the depletion of SLF, GC, or UGO at the tachyzoite stage. CDC50.1, UGO, and SLF are crucial for GC localization, whereas cGMP production of parasites depleted in CDC50.1 was only partially impaired (10). Although GC activity was not directly measured in this study, the block in microneme secretion and egress upon BIPPO stimulation suggests that cGMP production was abolished in SLF-depleted parasites, as observed in UGO-depleted parasites. Therefore, we conclude that SLF is critical for the assembly of the complex as well as GC activity. Moreover, SLF- and GC-deficient extracellular parasites displayed significantly reduced (but not null) conoid extrusion and F-actin basal accumulation upon BIPPO stimulation.

UGO is a large polytopic protein presenting a series of conserved TM domains at both N- and C-terminal protein regions and a central ectodomain unique to individual parasite species. The ectodomain of UGO was proposed to be involved in sensing of external signals (20). The Toll-like receptor signaling regulator (UNC93B1) has membrane transporter features and works as a chaperone of Toll-like receptors (56), resembling the activity of SLF, but its substrate is unknown. SLF could be a putative ion transporter or sensor, possibly involved in the sensing of known external signals (calcium, potassium, and proton levels) for the parasites’ programmed egress. SLF lacks conserved properties of calcium binding sites (the canonical EF hand and calmodulin-like domain), which makes the ability of SLF to deliver calcium unlikely. In the human synaptic vesicle, proton-coupled antiporters use low pH to drive neurotransmitters inside (57). The SLF knockdown strain was not responsive to acidic environment, as shown by a dramatic egress defect. However, this can be explained by either the loss of GC activity or the inability of SLF to sense low pH. Hence, recombinant expression of active SLF *in vitro* and construction of a mutation in which SLF activity is impaired but GC localization is unchanged would be critical to determine its contributions.

In Plasmodium yoelii, gametogenesis essential protein 1 (GEP1) containing 14 TMs is a possible sodium neurotransmitter symporter, showing some sequence similarity with SLF. GEP1 interacts with GCα, which is responsive for cGMP synthesis in gametogenesis. GEP1-deficient parasites abolish xanthurenic acid (XA)-stimulated and zaprinast (PDE inhibitor)-treated cGMP synthesis. The phenotype of GEP1 disruption mimicked that of GCα knockdown, leading to a block in gametogenesis and formation of exflagellation centers in male gametocytes. Of note, GEP1 depletion did not change the GCα expression in cytosol puncta of gametocytes, different from SLF disruption for GC mislocalization (58). No GEP1 homologue could be found in T. gondii.

Acute infection of T. gondii is the result of tachyzoite repetitive lytic cycles, which are tightly regulated by the GC. We showed by IFA that SLF is expressed in *in vitro* differentiated bradyzoites. GC is also likely expressed in bradyzoites based on RNA-seq data (ToxoDB). A recent study unveiled that although bradyzoites store less calcium than tachyzoites, the calcium signaling pathway plays a critical role in gliding motility of bradyzoites, as demonstrated by inhibition of key players (PKG and CDPK1). Bradyzoites within *in vivo* cysts became motile but failed to egress upon treatment with A23187 or zaprinast treatment (33). It seems that the thick cyst wall of *ex vivo* cysts restrict the bradyzoites’ ability to egress. *In vivo*, bradyzoites need to quickly invade intestinal epithelium cells after oral ingestion of tissue cysts. A prior study also has shown that *in vitro* bradyzoites from ruptured cysts can reinvade new host cells (59). To investigate if the ability of GC complex to control bradyzoite invasion, motility and/or egress contributes to bradyzoite cysts’ dissemination, we applied a promoter swap strategy to selectively knock down genes of the complex in bradyzoites. When controlled b*y* the *SAG1* promoter, SLF and GC were overexpressed compared to the endogenous level but did not induce any significant toxicity. In *in vivo* experiments, weight loss and survival of the mice can serve as a readout for virulence of each strain. For the intraperitoneal infection experiments with tachyzoites, sag1/SLF-Ty parasites presented a virulence pattern comparable to that of the ME49 parental strain. The sag1/Myc-GC parasites seemed less virulent based on a significant decrease of weight loss and the fact that no mice succumbed to intraperitoneal infection (Fig. 5A and B). In contrast, the oral gavage experiments with sag1/Myc-GC cysts elicited a significant increase in weight loss of mice and a decrease in mouse survival, compared to cyst infection with the ME49 parental strain (Fig. 6A and B). The long-term survival of mice is determined by both acute and chronic infection. During the acute stage (first 2 weeks) of both infections, survival of mice infected with either GC mutant or ME49 parental parasites presented no statistically significant difference (Fig. 5B and 6B). Of relevance, the fluctuations of parasite virulence exist due to the initial low-dose infection. Taken together, it is difficult to compare and to infer a change in virulence between the SLF and GC promoter swap tachyzoites *in vivo*.

Upon ME49 infection, the number of cysts surges, reaches a plateau in mouse brain before week 2 postinfection, and remains stable as the chronic phase progresses. This phenomenon reinforces the idea that efficient immune response of the host starts around 2 weeks after inoculation and enables to clear majority of bradyzoites from cyst rupture (60). The cyst burden in mice infected with both GC- and SLF-knockdown transgenic lines was reduced compared to that in parental strains at both time points examined, indicating that the GC complex contributes to the onset of chronic infection. Moreover, inoculation of 100 cysts or 10 cysts produced comparable numbers of cyst, probably indicating a bottleneck of cyst infection.

Rather unexpectedly, the cysts isolated from sag1/SLF-Ty or sag1/Myc-GC strains were still infectious in *in vitro-*cultured HFF cells. Stage differentiation of T. gondii normally needs 2 to 3 days (61). However, the environment of HFF cells may rapidly trigger tachyzoite conversion and breakdown of cyst wall, restoring higher levels of SLF and GC. In addition, it is worth mentioning that parasites inside one mature cyst are not homogenous in their stage differentiation. On many occasions, we could detect a minority of SAG1-positive parasites inside mature tissue cysts from mouse brain (Fig. 5C), consistent with SAG1-positive parasites detected in *in vitro* cysts (33).

Remarkably, when given to mice by the oral route, GC-depleted bradyzoites were still able to establish infection, whereas bradyzoites without SLF lost the capacity to infect mice. Compared with *in vitro* culture, the environment during oral infection differs in many ways. The pH stands out since gastric juice is highly acidic (pH 1.0 to 2.5) and the pH in the small intestine is around 6.0, while HFF culture is maintained at a pH 7.4. Tachyzoites are less resistant than bradyzoites to an acidic environment (62), and if the cysts liberated tachyzoites, they would survive better in culture than through the natural route of infection. Moreover, the mucosa of the small intestine that bradyzoites needs to penetrate consists of a variety of cells releasing mucins, metabolites, and hormones. The discrepancy between sag1/SLF-Ty or sag1/Myc-GC strains might also result from differential tightness of the promoter swap. Such a discrepancy was also observed in the downregulation of UGO, which led to a more severe depletion of basal cGMP than downregulation of GC itself (10). Therefore, a low basal level of cGMP in sag1/Myc-GC bradyzoites that would not be reached in sag1/SLF-Ty bradyzoites may be sufficient to ensure parasite invasion. Alternatively, and more interestingly, the lack of oral infectivity of sag1/SLF-Ty bradyzoites could reflect a unique and dedicated role of SLF in sensing the environment upstream of GC-mediated signaling in the intestine.

In *Trypanosomes*, a cAMP response protein, CARP3, is required for acid sensing *in vitro* and for establishment of infection in flies, highlighting the key role of cAMP signaling in the perception of pH taxis by trypanosomes (63). GC and SLF are predicted to be phosphorylated by protein kinase A (PKA) as well as CDPK3 (64), and disruption of CDPK3 leads to the elevation of cAMP, which tightly regulates PKA (26). Hence, cAMP might contribute to control of the GC activity in various environments.

In conclusion, the GC complex might perceive and transduce multiple signals when facing different environments, initiating a signaling cascade that is essential for the tachyzoite and bradyzoite lytic cycles, the latter contributing to the onset of chronic infection.

## MATERIALS AND METHODS

### Parasite strains.

The RH/Tir1 (Tir1) (65) and ME49 strains were used to construct genetically modified strains. Recipient strains and transgenic strains were all maintained in confluent human foreskin fibroblast (HFF) cells covered with culture medium that contains Dulbecco’s modified Eagle’s medium (DMEM) (Gibco), 5% fetal bovine serum (FBS), 25 μg/mL gentamicin, and 2 mM glutamine.

### Antibodies.

The sources of the primary antibodies used for IFA and western blotting are as follows: polyclonal rabbit anti-GAP45 (1:10,000) (66), anti-HA (1:1,000) (Sigma; H6908), anti-catalase (1:2,000), anti-SAG1 (1:2,000), anti-AMA1 (1:2,000), and anti-trimethyl lysine (1:3,000) (Immunechem; ICP0601); monoclonal mouse anti-actin (1:20) (67), anti-Ty (1:10, BB2), anti-GRA1 (1:20) (J. F. Dubremetz), anti-GRA3 (1:20) (68), anti-MIC2 (1:10), anti-P21 and anti-SAG1 (1:10) (BB2), and anti-GFP (1:1,000); and guinea pig anti-α-tubulin and anti-β-tubulin (1:250).

The secondary antibodies for IFA were Alexa Fluor 405-, Alexa Fluor 488-, and Alexa Fluor 594-conjugated goat anti-mouse, rabbit, or guinea pig antibodies (Life Technologies) and fluorescein isothiocyanate (FITC)-conjugated lectin (DBA, 1:500) (Vector Laboratories; FL-1031-2).

The secondary antibodies for western blotting were peroxidase-conjugated goat anti-rabbit or mouse antibodies (Sigma).

### DNA cloning and construction of genetically modified lines.

**(i) SLF-mAID-Ty and SLF-mAID-HA.** To produce auxin-inducible degradation of SLF, the PCR fragment containing the mAID tag and the HXGPRT selection was amplified from the vector pTUB1:YFP-mAID-3Ty or pTUB1:YFP-mAID-3HA using the primers 9914/9915 and KOD polymerase (Novagen, Merck). Primers 9914/9915 carry 30 bp of homology with the 3′end of SLF. A CRIPSR/Cas9 plasmid with a specific guide RNA (gRNA) targeting the 3’end of SLF was generated from pSAG1-Cas9-GFP-U6::sgUPRT plasmid (69) by a Q5 Hot Start site-directed mutagenesis kit (NEB) and primers 4883/9913 to induce double-strand DNA breaks and direct the insertion of the PCR fragment.

**(ii) Tagging: SLF-Ty, SLF-mAID-HA/GC-Ty, SLF-mAID-HA/UGO-Ty, GC-mAID-HA/SLF-Ty.** For the Ty tagging, we amplified a PCR fragment encoding 2 Ty epitopes and a dihydrofolate reductase (DHFR) cassette from the vector pLinker-2Ty-DHFR using KOD polymerase and primers of each gene. Primers 9914/9915, 7427/7428 and AIDfo-238390/AIDrev-238390 carry sequences with 30 bp of homology to SLF, GC, and UGO, respectively. A specific single guide RNA (sgRNA) was designed to direct Cas9 nuclease to break the C terminus of each gene. (The primers used to generate the guide RNA for SLF, GC, and UGO were 9913, 7426, and gRNA-238390, respectively). SLF-mAID-HA was the recipient strain for GC tagging and UGO tagging. SLF was Ty tagged in both the ME49 and GC-mAID-HA strains (10).

**(iii) Promoter swap strains: sag1/SLF-Ty and sag1/Myc-GC.** To generate SLF and GC promoter swap strains, SAG1 promoter cassette was first amplified from sag1-KOZAK-MYC-RFP-CAT plasmid (50) by KOD polymerase with primer pairs 10151/10152 and 10006/10007, respectively. These primers carry 40-bp homology sequences corresponding to the 5′ region upstream of predicted promoters of targeted genes and 3′ region after the start codon of targeted genes, respectively. CRISPR/Cas9 plasmids containing GFP and 2 gRNAs targeting predicted SLF and GC promoter regions were constructed as described before (70). Subsequently, 15 μg of KOD-amplified SAG1 promoter cassette and 20 μg of 2gRNA plasmid were precipitated using sodium acetate and then cotransfected into the SLF-Ty strain for sag1/SLF-Ty and the ME49 strain for sag1/Myc-GC. Without drug selection, parasites expressing GFP were sorted by fluorescence-activated cell sorter (FACS) analysis. The integration of SAG1 promoter cassette checked by primers 9515/10213 and 1935/10214 and loss of the predicted SLF promoter region checked by 10213/10215 indicate the successful generation of the sag1/SLF-Ty strain. Primers 9515/10056 and 1935/10057 were used to check the integration of Myc tag and *SAG1* promoter for GC. Primers 10056 and 10058 were subjected to PCR diagnosis of the replacement of GC promoter region.

All primers used in this study are listed in Table S1.

10.1128/mbio.01965-22.5 TABLE S1 Primers used in this study. Download Table S1, PDF file, 0.3 MB. Copyright © 2022 Ye et al. 2022 Ye et al. https://creativecommons.org/licenses/by/4.0/ This content is distributed under the terms of the Creative Commons Attribution 4.0 International license.

### Plaque assay.

A limited amount of freshly egressed parasites was inoculated into confluent HFF monolayers in the absence or presence of IAA and given 7 days of propagation for type I strains or 12 days of growth for type II strains without disturbance under standard conditions (37°C, 5% CO_2_).

To test the viability of *ex vivo* cysts, HFF cells were washed with media the day after the inoculation with cysts from brain homogenate. Plaques were visualized through 0.1% crystal violet (Sigma) staining.

### Intracellular growth assay.

Freshly egressed tachyzoites were inoculated into HFF cells embedded on coverslips. After 1 h of invasion, the parasites that had not invaded were washed away with phosphate-buffered saline (PBS), while the intracellular ones were allowed to grow under standard conditions in the presence or absence of IAA for another 24 h (for type I strains) or 30 h (for type II strains). Samples were fixed with paraformaldehyde (PAF)- glutaraldehyde (Glu) followed by IFA. Parasites were visualized by GAP45 staining. A total of 200 PVs were counted for each sample, and the experiment was repeated three times.

### Green/red invasion assay.

SLF-mAID-Ty and Tir1 were pretreated with or without IAA for 24 h. Parasites were released naturally or by syringe passage and then inoculated onto HFF monolayers embedded on coverslips. After a quick spin down, the parasites were allowed to invade at 37°C for 30 min. Subsequently, IFA was performed to visualize and distinguish invaded and noninvaded parasites. In brief, extracellular parasites were labeled with anti-SAG1 antibodies prior to the permeabilization of cells. After washing away the SAG1 antibodies, cells were fixed with PAF-Glu and permeabilized with 0.2% Triton X-100–PBS. All parasites were then labeled with anti-GAP45 antibodies. Noninvaded and invaded parasites were distinguished by staining with Alexa Fluor 488- (green) and Alexa Fluor 594 (red)-conjugated goat anti-mouse or anti-rabbit antibodies. At least 100 parasites were counted for each stain. Data were collected from three independent biological experiments.

### Egress assay.

Freshly egressed parasites were inoculated into confluent HFF cell-coated coverslips and treated with or without IAA for 30 h. After being washed with serum-free medium, the cells were incubated in serum-free medium containing egress inducers (10 μM BIPPO, 3 μM A23187, or 100 μM propranolol) or DMSO at 37°C for around 10 min. For low-pH-induced egress, after three washes with intracellular (IC) buffer (5 mM NaCl, 142 mM KCl, 1 mM MgCl_2_, 2 mM EGTA, 5.6 mM glucose, 25 mM HEPES, with pH adjusted to 7.2 with KOH), the cells were incubated with IC buffer with 7.5 μM digitonin and at pH 7.2, 6.2, or 5.2 (10, 11). Cells were then fixed with PAF-Glu and quenched in glycine-PBS. IFA was performed to label parasites with GAP45 antibody and the PV membrane with GRA3 antibody. At least 100 vacuoles were observed to calculate the proportion of egressed and nonegressed vacuoles. Results are presented as the mean ± standard deviation (SD) from three independent replicates.

### Microneme secretion.

Confluent HFF cells were heavily infected with freshly egressed parasites and covered with or without IAA. When the majority of parasites naturally egressed, 2 × 10^7^ parasites from each strain were harvested. Parasite pellets were washed with IC buffer and then resuspended in 100 μL warm serum-free medium containing egress inducer (2% ethanol, 10 μM BIPPO, or 50 nM ePA). After 30 min of incubation at 37°C, parasites were centrifuged at 4°C. Subsequently, the pellet was washed with PBS, while the supernatant (ESA) was cleared again via a second centrifugation. The pellet and ESA were subjected to immunoblotting. MIC2, as a representative microneme, was detected by its secretion and processing. GRA1 and catalase are markers for constitutive secretion and parasite lysis. The same results were observed from three independent biological repeats.

### Gliding/trail motility.

SLF-mAID-Ty and Tir1 parasites were pretreated with or without IAA for 24 h. One hundred microliters of fresh parasites extracted from a 3-cm dish was pipetted on coverslips coated with 0.1% gelatin. Subsequently, parasites were incubated with 500 μL of warm serum-free medium containing 10 μM BIPPO at 37°C for 15 min. After fixation with PAF/Glu, IFA without Triton X-100 was performed on coverslips using anti-SAG1 antibody to visualize parasite gliding trails. The same results were observed from three independent experiments.

### Conoid extrusion assay.

Extracellular parasites from a 3-cm dish were pelleted and then incubated in 300 μL of warm PBS with 10 μM the extrusion trigger BIPPO at 37°C for 15 min. Thereafter, U-ExM was conducted as previously described (47). Fifty microliters of parasites was sedimented on 12-mm coverslips coated with poly-d-lysine during 15 min at room temperature. After this, the coverslips were transferred into a 12-well plate and bathed in 1 mL 1.4% formaldehyde–2% acrylamide (FA/AA)-PBS solution for prevention of protein cross-linking and addition of anchors to each free amine group. Five hours later, a drop of ammonium persulfate (APS)-TEMED-monomer solution (19% sodium acrylate, 10% AA, 0.1% methylenebisacrylamide in PBS) was rapidly covered with a coverslip with a parasites’ facing solution. The gelation step lasted for 30 min at 37°C to allow parasite proteins to be tethered to the gel mesh. Gels coupled with parasite proteins were then denatured at 95°C for 1.5 h. After the first round of expansion with water, gels were shrunk in PBS to prepare them for immunostaining. A 3-h incubation of gels with guinea pig anti-α-tubulin and anti-β-tubulin in PBS–2% bovine serum albumin (BSA) was performed. Subsequently, primary antibodies were detected by Alexa 594-conjugated goat-guinea pig IgG secondary antibodies. Before imaging, gels were subjective to second round of expansion. Finally, images were acquired with a Leica TCS SP8 inverted microscope with maximum projections and processed with ImageJ. Inverted fluorescence microscopy (Olympus) with a 100× lens objective was also allowed to distinguish conoid nonextruded and conoid extruded parasites and used for counting (with a minimum of 100 parasites analyzed). SLF-mAID-Ty and Tir1 were treated with or without IAA 24 h prior to the assay.

The extruded conoid is also clearly detectable by staining methylated preconoidal rings and the apical cap of parasites (8). Tir1 parasites were pretreated with or without 1 μM cytochalasin D (CD) (an inhibitor of actin polymerization) (44) or with or without 1 μM compound 1 (C1) (a PKG inhibitor) (71) for 30 min. After this, 50 μL of fresh extracellular parasites was added on gelatin-coated coverslips, which were subsequently incubated with 100 μL of warm egress medium with or without BIPPO for 15 min. Thereafter, the coverslips were fixed with PAF-Glu and then subjected to IFA using pan-*N*-ε-trimethyl lysine and antiactin antibodies. A separated apical dot stained with pan-*N*-ε-trimethyl lysine antibody signifies extruded preconoidal rings, which also indicates conoid extrusion. BIPPO-induced conoid extrusion was examined among a minimum of 100 parasites in an individual experiment.

### F-actin basal accumulation.

Parasites were transiently transfected with pT8-chromobody-GFPTy. After transfectants were treated with or without IAA for 48 h, freshly egressed or syringe-lysed parasites were placed on gelatin-coated coverslips and then treated with 10 μM BIPPO for 15 min at 37°C/5% CO_2_ to induce actin flux. The coverslips were fixed with PAF-Glu and subsequently subjected to immunostaining using GFP and AMA1 antibodies. A basal dot of GFP staining indicates F-actin basal accumulation. At least 100 parasites were checked for each experiment. The quantitative result was obtained from three independent experiments.

### Mouse infections.

All animal experiments were conducted under authorization no. GE121-19 and GE-125 according to the guidelines and regulations issued by the Swiss Federal Veterinary Office.

Six-week-old female B6CBAf1 mice (Janvier labs) were intraperitoneally injected with 150 freshly egressed tachyzoites in 200 μL DMEM. Mice were monitored daily and sacrificed when they showed severe signs of infection (ruffled fur, difficulty moving, isolation, and/or excessive weight loss). Surviving mice were sacrificed at 2 or 6 weeks postinfection to determine cyst burden and mouse serum was collected for seroconversion analysis.

After 6 weeks of intraperitoneal infection, brains were collected from chronically infected mice and homogenized by sequential passaging with needles. For oral infection, cysts from 6-week chronically infected mice were quantified within 2 days of the collection. Ten or 100 *ex vivo* cysts in 200 μL sterilized PBS were given to each mouse by oral gavage.

### Tissue cyst purification, counting, and size measurement.

When mice were sacrificed, mouse brains and blood were collected. After a PBS wash, mouse brains were resuspended in 1 mL PBS and smashed using syringe passages (18G 15 times, 21G 10 times, and 23G 5 times).

Five hundred microliters of brain homogenate from each mouse was fixed with 500 μL of 8% PAF for 30 min. After being centrifuged at 2,000 × *g* for 5 min, brain mixtures were then quenched with 1 mL PBS-glycine for 5 min. After permeabilization and blocking, brain homogenate was incubated with primary antibody (rabbit anti-GAP45) in Triton X-100–BSA for 1 h followed by DBA and secondary antibody (Alexa Fluor 594-conjugated goat anti-rabbit) staining. After one Triton X-100–PBS wash, brain homogenate was resuspended in 50 μL Triton X-100–PBS. Cysts with GAP45 and DBA staining were counted in duplicate (10-μL aliquot) by inverted fluorescence microscopy, and the diameters of DBA signal were measured with the NIS-element C-ER system.

### Statistical analysis.

Statistical analyses were performed in Prism 8 (GraphPad Software, Inc., La Jolla, CA, USA) using a two-tailed Student's *t* test, a two-tailed Mann-Whitney test, or the logrank Mantel-Cox test, as indicated in the figure legends.
