# Supplementary figures and images for: A Signaling Factor Linked to Toxoplasma gondii Guanylate Cyclase Complex Controls Invasion and Egress during Acute and Chronic Infection
Source: mBio. 2022 Oct 6;13(5):e01965-22. doi: 10.1128/mbio.01965-22 (PMC9600588; doi:10.1128/mbio.01965-22)

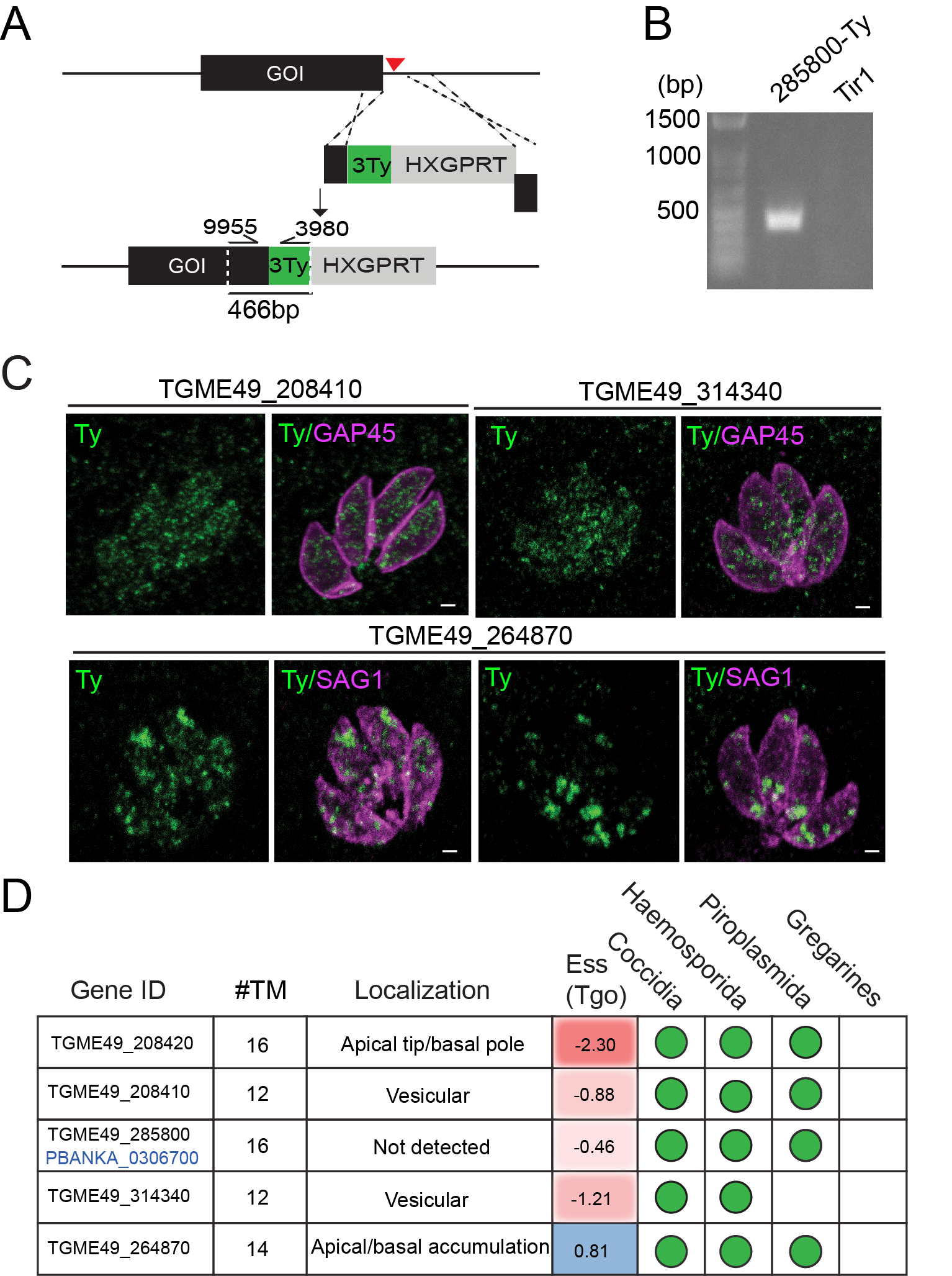

Supplement: FIG S1 [file mbio.01965-22-s0001.tif]

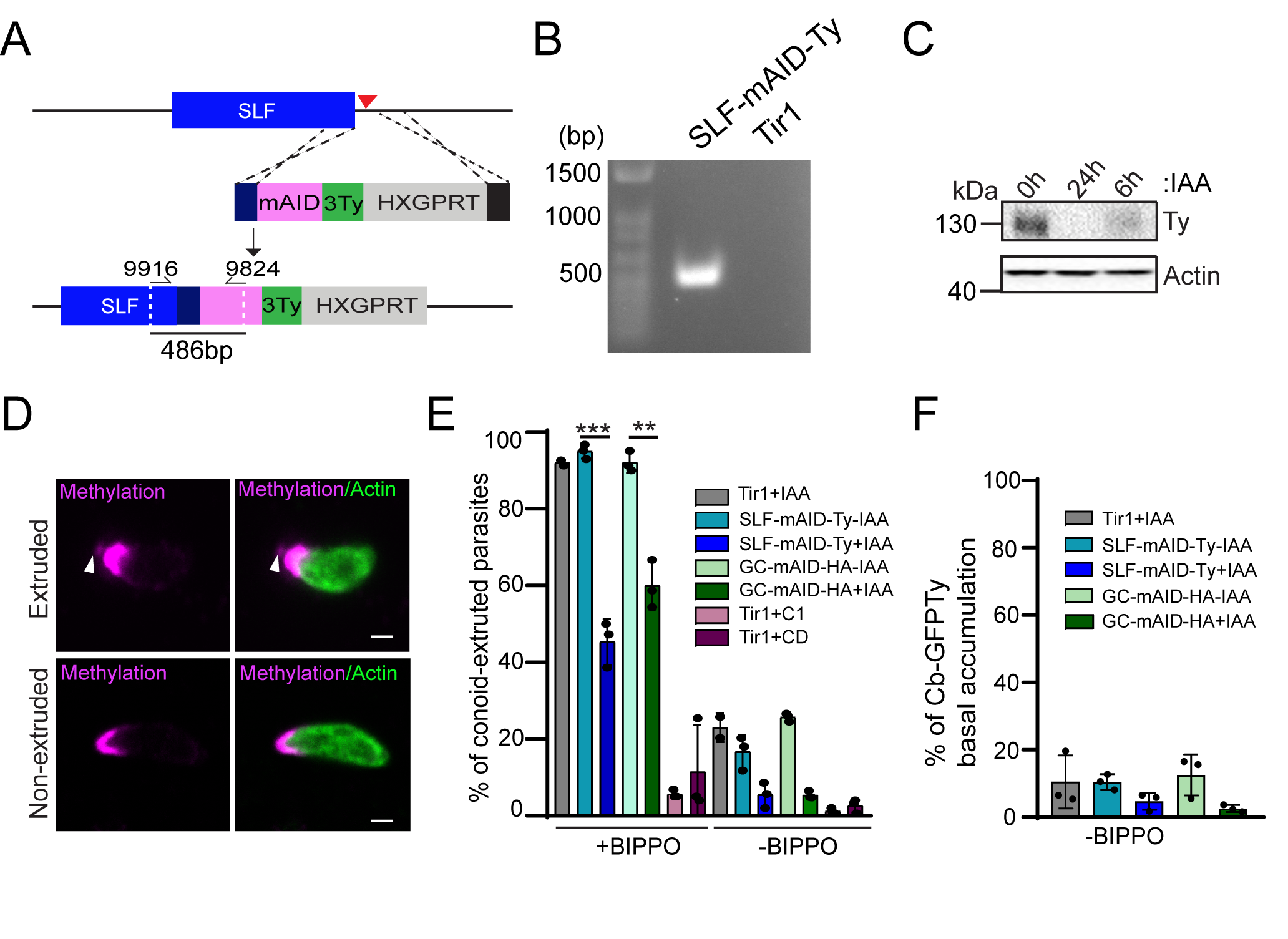

Supplement: FIG S2 [file mbio.01965-22-s0002.tif]

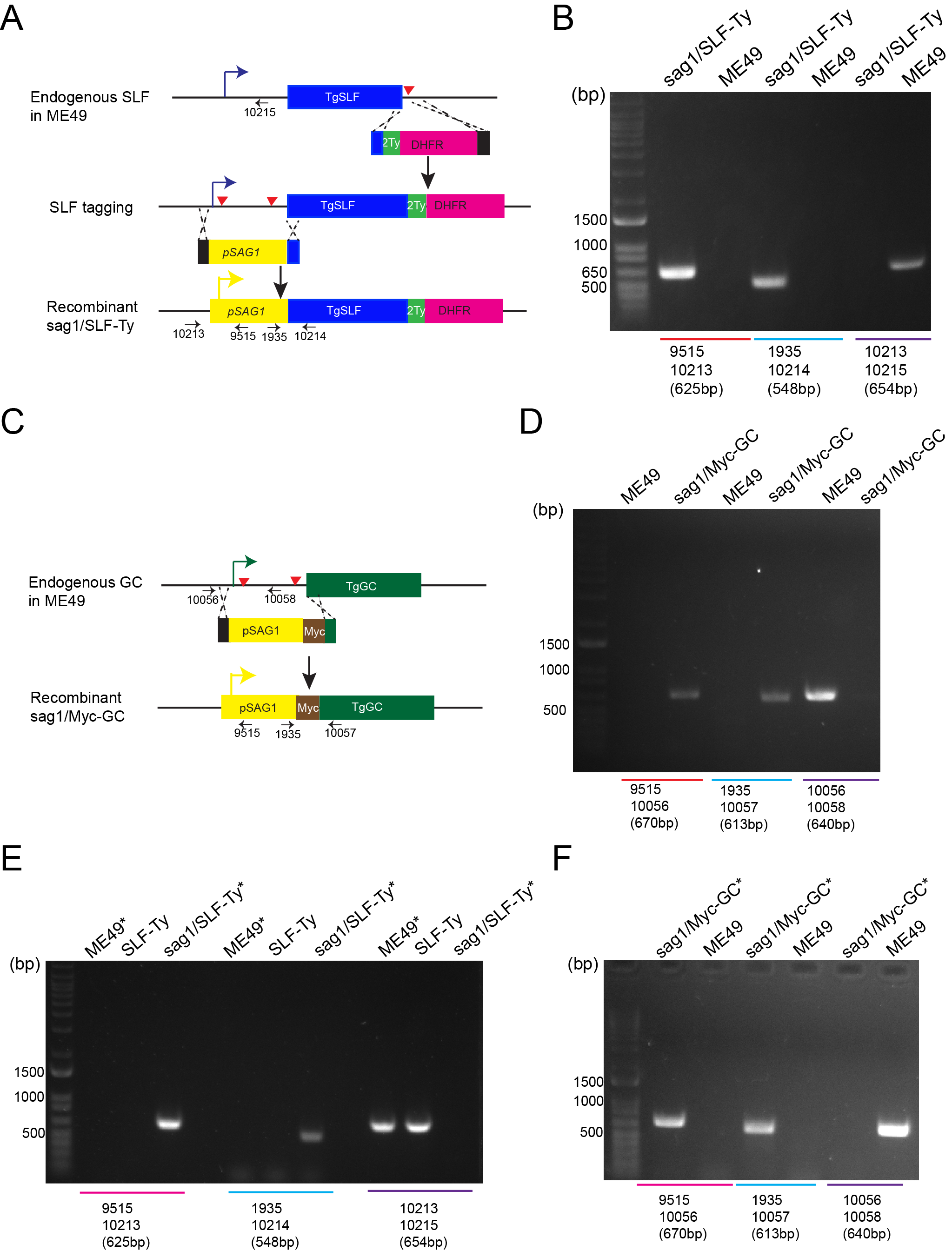

Supplement: FIG S3 [file mbio.01965-22-s0003.tif]

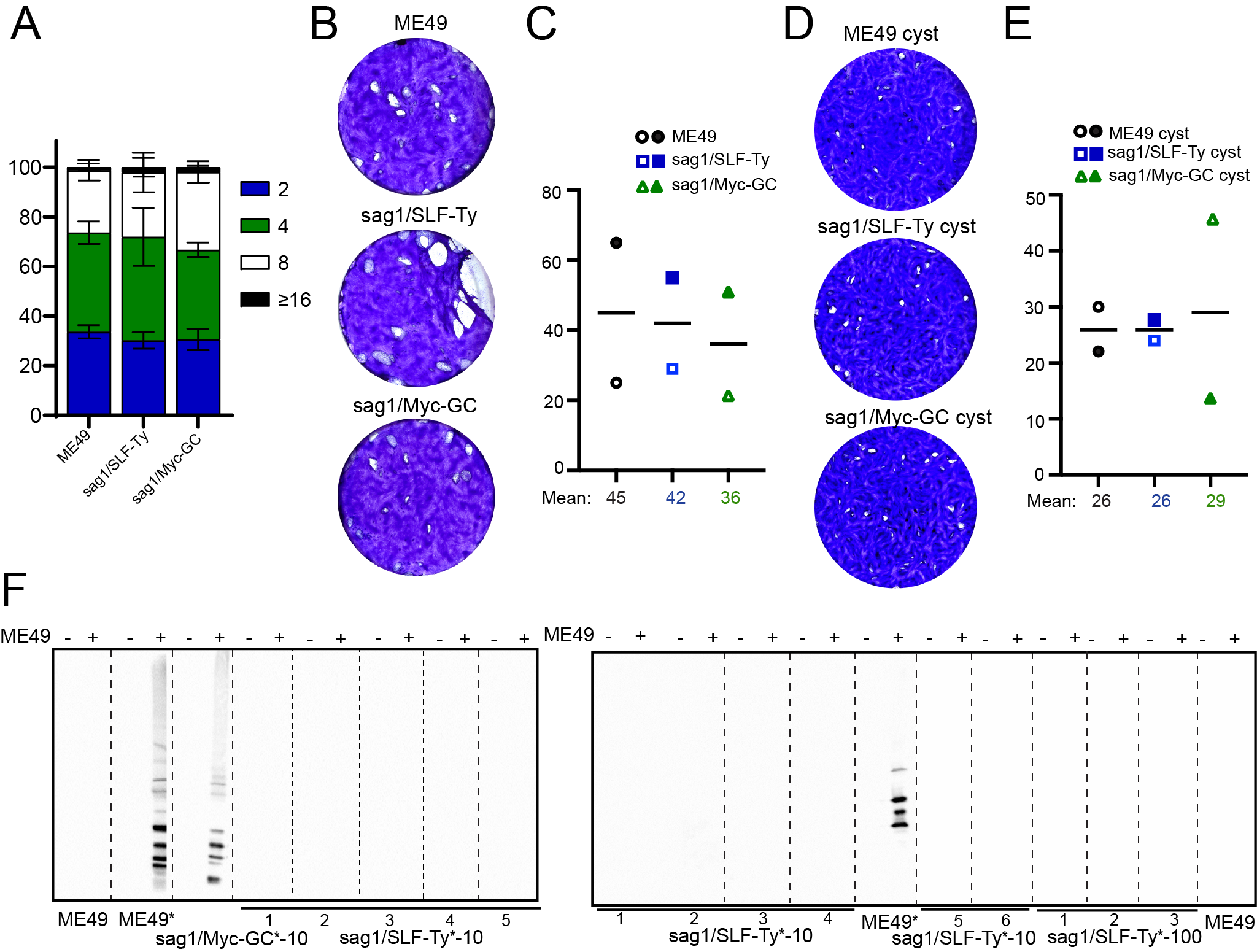

Supplement: FIG S4 [file mbio.01965-22-s0004.tif]
