# Supplementary material for: A Signaling Factor Linked to Toxoplasma gondii Guanylate Cyclase Complex Controls Invasion and Egress during Acute and Chronic Infection
Source: mBio. 2022 Oct 6;13(5):e01965-22. doi: 10.1128/mbio.01965-22 (PMC9600588; doi:10.1128/mbio.01965-22)
Supplement: TABLE S1 [file mbio.01965-22-s0005.pdf]

Table S1. Primers used in this study

Primers	Number	Sequence(5'-3')	Use
gRNA-Rv	4883	AACTTGACATCCCCATTTAC	To construct CRISPR/Cas9 plasmid with one gRNA
SLF-gRNA	9913	GTTTAACCTCATGCACCAACGGTTTTAGAGCTAGAAATAGC	To construct CRISPR/Cas9 plasmid with gRNA targeting SLF C-terminus
SLF-mAID-Fw	9914	TATGTTTTCTCTGTCGCAGCCTCCAAGTCTGCTAGCAAGGGCTCGGG	To generate SLF-mAID cKD/HXGPRT selection and SLF-2Ty DFHR selection
SLF-mAID-Rv	9915	TTTCCAAACCACAATCGAAGCTCTCTAAACATACGACTCACTATAGGG	
In-SLF-Fw	9916	GCGGTTCGTGTAGAGCGG	To check integration in SLF
SLF-SAG1-Fw	10151	CGGTGAAGGTTCTGTCCGGGACCGGCCGATATAGATGGACGCAACGCGTGTCACAAGC	To PCR amplify SAG1 promoter with 40bp SLF homology
SLF-SAG1-Rv	10152	CGGATTTCTGCCGAAACCTGGTCGTGTACAATCGCTTCATCATTTTGCCTCTCCTGCAGG	
SLF-2gRNA-1#	10149	GTAAATGGGGATGTCAAGTTGGCAAATCTTTCGTTCCACTGGTTTTAGAGCTAGAAATAGC	To construct CRISPR/Cas9 plasmid with 2gRNA targeting SLF original promoter region
SLF-2gRNA-2#	10150	GCTATTTCTAGCTCTAAAACTCTGCTCTCACACTTCTAGCCAACTTGACATCCCCATTTAC	
5’-UpU5SLF	10213	CAGTAACTCGGCGGCAAAAC	To check the integration of 5’ SAG1 promoter
SAG1-pro-Rv	9515	CCGTGGAAAAGGCAACGG	
SAG1-pro-Fw	1935	CGCTGCACCACTTCATTATTTCTTCTGG	To check the integration of 3’ SAG1 promoter
In-SLF-Rv	10214	GGAACAGATGGCTGACGGTG	
SLF-pro-Rv	10215	CGCAAAGACTGGGCAGAATG	To check the presence of original SLF promoter region
GC-mAID-Fw	7427	GGGTCGACACCTGGCTCTGCACTCGGGTCGGCTAGCAAGGGCTCGGG	To generate GC-2Ty/DHFR selection
GC-mAID-Rv	7428	ACTGCCCGAAGCGGCAGGACACAGACCCGCATACGACTCACTATAGGG	
GC-gRNA	7426	GTCTGGAGCAACGCAGAACCTGTTTTAGAGCTAGAAATAGC	To construct CRISPR/Cas9 plasmid with gRNA targeting GC C-terminus
AIDFw-238390		GGCTCTGGCCACCTTGTCGGCGATCGTAGCGCGGCTAGCAAGGGCTCGGG	To generate UGO-mAID cKD/HXGPRT selection and generate UGO-2Ty/DHFR selection
AIDRv-238390		ACAGACACACACGCATACCCAAACGTGCAGATGATACGACTCACTATAGG	
gRNA-238390		GAGAGCTTGTCAGCATGGAGTGTTTTAGAGCTAGAAATAGC	Guide for UGO cKD and tagging
GC-SAG1-Fw	10006	TTGCTCCTCTTCGCCTTCCACCTGCTGTCCCTGTTTGTCCGCAACGCGTGTCACAAGC	To PCR amplify SAG1 promoter with 40 bp of GC homology and Myc tag
GC-SAG1-Rv	10007	AATCCGGTGCTGTTCAGGTTGTCTTACTTCGCGTCTTCTTCAGGTCCTCCTCGGAGATG	
GC-2gRNA-1#	10017	GTAAATGGGGATGTCAAGTTGGAGGCCAGAAATATCAAGACGTTTTAGAGCTAGAAATAGC	To construct CRISPR/Cas9 plasmid with 2gRNA targeting GC original promoter region
GC-2gRNA-2#	10018	GCTATTTCTAGCTCTAAAACCTGTCTTCTATTCCGGAGCTCAACTTGACATCCCCATTTAC	
5’-UpU5GC	10056	CGAGCTCCCCGTTTCTTTCC	To check the integration of 5’ SAG1 promoter
In-GC-Rv	10057	CCGAAAAGCCTGACTCTCCC	To check the integration of 3’ SAG1 promoter
GC-pro-Rv	10058	GACAAAGGGAACCACGGGTG	To check the presence of original GC promoter region
gRNA-208410	9917	GAAAAGATCAGCAAAACCCAGGTTTTAGAGCTAGAAATAGC	gRNA targeting Cterm of TGME49_208410
208410	9918	TCCGGAAGTGGAGGCCCCGAATCAGAAGGGCGCCCCGCGCGCGATGCA	To generate TGME49_208410 tagging strain/HXGPRT selection
208410	9919	GACCCTTGGAACAGTCCTAGGTGTTTCAGGGCTGGAGCTCCACCGCGG	
285800	9920	GCCACGCGAGAGAGGAAGCCAGTTTTAGAGCTAGAAATAGC	Guide for ME49_285800 tagging
285800	9921	CACTGGAAGGTTGCGACTGACGAACTCGCCCGCCCCGCGCGCGATGCA	To generate TGME49_285800 tagging strain/HXGPRT selection
285800	9922	CTTCGGACGAGGTCTCTTGCACCCAAATCCGCTGGAGCTCCACCGCGG	
285800	9955	AGAACAGTTTCCGGTCGGC	To check the integration of ME49_285800 tagging with 3Ty
285800	3980	GTGACACCTGCAAGCCACAGCGG	
314340	9923	GTGAGTAGGTTGAGGACCGAAGTTTTAGAGCTAGAAATAGC	Guide for ME49_314340 tagging
314340	9924	GGGGACAAGTCTCCGACTCAATCGAGGCAGCGCCCCGCGCGCGATGCA	To generate TGME49_314340 tagging strain/HXGPRT selection
314340	9925	TCGCAAAAGCGCACTCCGATCCACTGCATGGCTGGAGCTCCACCGCGG	
264870	9926	GAGTGCGAGGCTTGAGAACCTGTTTTAGAGCTAGAAATAGC	Guide for ME49_264870 tagging
